# Perceptions, attitudes, and concerns of underrepresented groups regarding the use of big data in children and young people’s healthcare research – A systematic literature review

**DOI:** 10.1371/journal.pdig.0001434

**Published:** 2026-10-15

**Authors:** Charlotte Burdge, Anne-Marie Burn, Lily Zhang, Sasha Menon, Karolina Kaminska, Sophie Wallace-Hanlon, Maria Fischer, Camille Evans, Peter Fonagy, Anna Wiedemann, Jessica Young, Anna Moore, Joanna K. Anderson

**Affiliations:** 1 Department of Psychiatry, University of Cambridge, Cambridge, United Kingdom; 2 Anna Freud, London, United Kingdom; 3 Cambridgeshire and Peterborough NHS Foundation Trust, Cambridge, United Kingdom; 4 Cambridge University Hospitals NHS Foundation Trust, Cambridge, United Kingdom; 5 Research Department of Clinical, Educational and Health Psychology, University College London, London, United Kingdom; National Tsing Hua University, TAIWAN

## Abstract

Children and young people (CYP), and those from underserved groups, remain underrepresented in biomedical research. Understanding their perspectives on large-scale, data-driven healthcare research is essential to ensuring that such approaches are acceptable, ethically conducted, and equitable. This review is the first to systematically examine the perceptions, attitudes, and concerns of underrepresented groups regarding the use of big data in CYP healthcare research. Seven bibliographic databases, ASSIA, CINAHL, EMBASE, MEDLINE, PsycINFO, Scopus, and Web of Science, were searched alongside grey literature sources. A mixed-methods systematic review design was used. Included studies captured the views of CYP, particularly those from underserved groups, as well as their parents or carers, acting as proxy decision-makers, regarding the use of big data in paediatric healthcare research. Study quality was appraised using the Mixed Methods Appraisal Tool (MMAT), and findings were integrated through narrative synthesis. Seventeen studies were included, capturing perspectives from 6,261 participants, including at least 4,710 CYP and 1,443 parents and carers; one study did not report participant numbers separately by group. Underrepresented groups expressed broadly favourable, but conditional, support for the use of big data in paediatric healthcare research. Willingness to share data was shaped by participant characteristics, lived experience, and trust in institutions. Perceived benefits included societal and public health gains, the opportunity to empower others with similar experiences, and the advancement of scientific progress. Concerns centred on privacy, the risk of re-identification, data misuse, commercial exploitation, and limited transparency in data governance. Conditions for acceptable data use included robust anonymisation, independent ethical oversight, and developmentally appropriate consent processes. Underrepresented groups hold broadly supportive, but conditional, views on the use of big data in paediatric healthcare research. Embedding inclusive governance practices and engaging these communities in research design will be essential to ensuring that data-driven paediatric research is both ethical and equitable.

## Author summary

Large-scale health datasets, often called *“big data”*, are increasingly used in medical research to identify patterns in disease, treatment, and healthcare use. Despite this, little is known about what children and young people (CYP), particularly those from underserved groups, think about how their health data should be used in research. We conducted a systematic review to address this gap, focusing specifically on the use of big data in CYP healthcare research. We found that CYP, parents, and carers were broadly supportive of data-driven healthcare research, but only under certain conditions. They raised important concerns about privacy, the risk of being identified from their data, and the possibility of data being misused, particularly by commercial organisations. They also recognised the potential for this research to benefit society and help others with similar experiences. Support for data use was greater where clear safeguards were in place, including strong privacy protections, independent oversight, and meaningful involvement in decisions about how data are used. Our findings highlight the importance of listening to underrepresented groups, who have historically had limited influence over research design and implementation, to ensure that future data-driven research is conducted ethically and that its benefits are distributed equitably.

## Introduction

Large-scale health datasets are transforming healthcare research. Drawn from electronic health records (EHRs), administrative systems, registries, and digital technologies, these data are increasingly analysed at scale to generate insights into disease patterns, treatment outcomes, and population health trends [1–4]. Used well, big data can also help us understand structural and institutional drivers of health inequity in ways that conventional clinical research cannot [5,6]. However, such approaches raise a distinctive set of ethical and social challenges: data are routinely reused beyond their original purpose, governance structures are often opaque, and participants are typically far removed from researchers who analyse their information. These features make trust, voice, and representation especially important. Studies of public attitudes to health data use have found broad but conditional support for data sharing, stronger where there is perceived public benefit and trust in the organisations involved [7–12]. Yet almost all of this evidence comes from general adult populations. The perspectives of children and young people (CYP), and of groups historically underserved by research and healthcare systems, remain largely absent. Structural disadvantage, proxy decision-making, and histories of institutional exclusion may shape attitudes in ways that general-population findings do not capture.

CYP’s perspectives are distinctive for reasons that go beyond their underrepresentation in research. Parents, carers, and guardians commonly advocate on their behalf, meaning CYP’s own views on healthcare and research participation are frequently overlooked in favour of proxy voices [13]. Proxy perspectives matter, but they cannot substitute CYP’s own attitudes and concerns, particularly in data-driven research, where sensitive longitudinal information may be reused or linked in ways neither participants nor proxies can fully anticipate. As children mature, they develop both the capacity and the desire to participate in decisions about their healthcare and personal data, a right recognised under Article 12 of the United Nations Convention on the Rights of the Child (UNCRC) [14,15]. Yet the systems through which health data are collected, governed, and reused rarely reflect this. Understanding what CYP themselves think about big data in healthcare research is not therefore a matter of inclusion for its own sake; it is a prerequisite for research that is ethically grounded in the people it most directly concerns [16].

CYP are not the only group whose perspectives are underrepresented in paediatric research. The National Institute for Health and Care Research **(**NIHR) characterises underserved groups as populations with *‘lower inclusion in research than one would expect from population estimates, high healthcare burden that is not matched by the volume of research designed for the group, or differences in how a group responds to or engages with healthcare interventions’* [17]. These groups are structurally and socially distinct, positioned differently in relation to healthcare institutions, facing different barriers, and holding different stakes in how their data are used. They should not be treated as homogeneous. Barriers to participation are multifaceted, spanning logistical challenges such as transport difficulties, time constraints, and financial pressures [17–21], as well as deeper structural and relational obstacles. Among these barriers, those rooted in mistrust of research and healthcare institutions may be especially significant in the context of data-driven research. Historical ethical violations in healthcare [22–24], alongside more recent instances of data misuse [25,26], have contributed to mistrust in healthcare research and healthcare systems, particularly among communities with lived experience of marginalisation or discrimination [27–31]. Where trust is absent, individuals may be less willing to participate in research or share personal health information [20,27,32], with direct consequences for the diversity and representativeness of health datasets. These dynamics are not uniform across groups. Intersecting identities can reinforce structural disadvantage and shape how individuals weigh the risks and benefits of data sharing in ways that vary considerably by context and experience [21,33].

Data do not simply reflect the world; they are shaped by the systems, research protocols, and institutional practices through which they are generated [5]. Communities that are historically underserved are therefore also likely to be underrepresented in datasets, and without their meaningful inclusion, data-driven research risks reproducing or amplifying the very inequalities it has the potential to address [34]. These dynamics are particularly acute for CYP from underserved groups, who may simultaneously navigate proxy decision-making, structural disadvantage, and histories of institutional exclusion. Ensuring big data research is both inclusive and equitable therefore requires not only representative datasets, but a genuine understanding of how communities perceive and experience the use of health data [5]. Patient and Public Involvement, Engagement, and Participation (PPIEP) has increasingly been recognised as a key mechanism for ensuring research reflects the priorities, concerns, and lived experiences of the communities it seeks to serve [35]. Yet for CYP and underserved groups, meaningful engagement remains the exception rather than the rule [13,16,36–38]. Despite growing recognition of the ethical and social dimensions of health data use, we know little about what these groups actually think, about who should have access to their data, under what conditions, and to what ends. This review addresses that gap directly.

### The current review

This systematic review examines how underrepresented groups perceive the use of big data in paediatric healthcare research, and the conditions under which they find it acceptable. Drawing on quantitative and qualitative evidence, it pursues three aims: a) to explore how perceptions and attitudes towards health data use vary across different underrepresented groups; b) to identify the concerns these communities hold about data sharing and research participation; and c) to examine what shapes their acceptance or rejection of data-driven approaches. Together, these aims reflect a broader commitment to ensuring that big data research is not only technically robust, but grounded in the perspectives of the people it most directly affects.

## Materials and methods

### Design

This systematic review was conducted in accordance with the PRISMA guidance [39] (S1 Checklist). The protocol was registered with PROSPERO on Feb 3, 2025 (CRD42025638882; PROSPERO International Prospective Register of Systematic Reviews). The JBI methodological guidance for the conduct of mixed-methods systematic reviews was followed [40]. Because the perspectives of underrepresented groups were captured using both quantitative and qualitative methods, a convergent integrated approach was used to synthesise and integrate the data.

### Search strategy

A comprehensive search strategy was developed (S1 Text), with input from PPIEP leads. The following bibliographic databases were systematically searched: ASSIA, CINAHL, EMBASE, MEDLINE, PsycINFO, Scopus, and Web of Science. MeSH terms and relevant text words in title and abstracts were used. Boolean operators were applied, with “OR” to combine search terms within each concept and “AND” to link across concepts. Search terms were informed by the NIHR-INCLUDE framework [17] and supplemented with additional keywords to capture underserved populations not explicitly listed in the framework, including CYP with chronic illness. Predefined search strategies developed by the Canadian Health Libraries Association [41] and the University of Alberta [42,43] were adapted to optimise identification of literature relating to CYP and underserved populations (S2 Text). Grey literature searches were also conducted (S3 Text), and the reference lists of included studies and relevant systematic reviews were hand-searched to identify additional eligible studies.

### Selection criteria

Articles published from database inception to Feb 18, 2025, were retrieved from all databases except ASSIA; ASSIA records were retrieved on Feb 20, 2025. No geographical restrictions were applied, and studies were deemed suitable for screening if they were reported in English. The SPIDER framework (Sample, Phenomenon of Interest, Design, Evaluation, Research type) was used to develop the inclusion and exclusion criteria [44]. Eligibility criteria for each SPIDER domain are described below and presented in Table 1.

**Table 1:** Eligibility criteria.

SPIDER Domain	Eligibility criteria	
	Inclusion	Exclusion
Sample	Underrepresented groups including CYP (aged 0–25 years). Parents, carers, or guardians where perspectives related specifically to underserved CYP.	Healthcare or research professionals. Parents, carers, or guardians reporting views on CYP broadly. Studies in which ≥50% of participants were not underserved CYP or their proxies.
Phenomenon of Interest	Perspectives on the use of big data for CYP healthcare research, including EHRs, administrative data, and linked datasets, encompassing data sharing, linkage, governance, and research participation.	Studies using big data solely for epidemiological or causal analyses without examining participant perspectives.
Design	Original empirical qualitative, quantitative, or mixed-methods studies, including grey literature.	Editorials, commentaries, opinion pieces, and non-empirical publications.
Evaluation	Perceptions, attitudes, concerns, trust, or views regarding big data use in CYP healthcare research, including barriers and facilitators influencing willingness to participate.	
Research type	Qualitative, quantitative, and mixed-methods studies reporting participant perspectives.	

#### Sample.

Studies were eligible if they examined the perspectives of underrepresented groups on the use of big data in CYP healthcare research. Perspectives could be reported directly by CYP, defined as individuals aged 0–25 years in line with the UK’s National Health Service (NHS) guidance recognising young people up to 25 years of age [45], or by parents, carers, or guardians acting as proxy decision-makers in relation to the use of CYP healthcare data. Studies reporting parental or caregiver perspectives were included only when these perspectives related specifically to underserved CYP populations rather than to CYP populations more generally. Underserved groups were defined in line with the NIHR-INCLUDE framework [17] as *‘populations whose inclusion in research is lower than expected relative to their population prevalence or healthcare burden’*. The framework was used to guide identification of underserved groups rather than to assume shared or uniform perspectives within them. We recognise that whether a population is considered underserved is context-dependent; eligibility was therefore determined through application of the predefined inclusion criteria rather than by search terms alone. Data-sharing attitudes may arise from diverse social and historical experiences (e.g., racism, stigma, illness burden, prior research contact, family dependence, fear of criminalisation, or distrust of commercial institutions) and therefore did not treat these groups as representing unified viewpoints. Studies focused exclusively on the perspectives of healthcare professionals or researchers were excluded.

#### Phenomenon of Interest.

Big data in healthcare research was defined as large-scale healthcare datasets used for research purposes, including EHRs, administrative health data, and linked healthcare datasets. This definition is consistent with descriptions of big data in healthcare as large, complex electronic health datasets [46]. This broad definition reflects the diversity of the big data landscape and encompasses heterogeneous data contexts that differ ethically, socially, and psychologically. For example, secondary use of EHRs, prospective donation of biological samples, linkage of administrative datasets, and use of mental health or smart device data for research may raise different questions relating to privacy, consent, identifiability, governance, and the perceived sensitivity of the data. We acknowledge that participant attitudes may partly reflect differences in the specific data contexts considered. The review therefore included perspectives on data sharing, linkage, governance, and research participation across these contexts, whether based on direct experience, hypothetical scenarios, or general discussion.

#### Design.

Studies reporting original empirical data using qualitative, quantitative, or mixed-methods designs were eligible. This included grey literature, including, but not limited to, organisational websites, conference proceedings, government and policy documents, and preprint databases such as medRxiv and PsyArXiv. Editorials, commentaries, opinion pieces, and other non-empirical publications were excluded.

#### Evaluation.

Studies were included if they explored perceptions, attitudes, concerns, trust, acceptability, or views regarding the use of big data in healthcare research. This included studies identifying barriers and facilitators that might influence willingness to share health data or participate in research involving big data.

#### Research type.

Qualitative, quantitative, and mixed-methods studies reporting participant perspectives were eligible.

### Study selection and data extraction

Peer-reviewed records retrieved from all databases were imported into Covidence [47] for deduplication and screening against the eligibility criteria. Screening was undertaken in two stages: title and abstract screening followed by full-text screening. Two authors (CB/LZ) screened a subset of articles (*n* = 50) at the title and abstract stage and achieved substantial agreement [48]. Disagreements were discussed with a third researcher (AW) to reach consensus. Additional authors (KK/MF/SWH) supported screening at both the title and abstract and full-text stages. Weekly meetings were held to review discrepancies and reach consensus across the wider group.

Grey literature sources were searched using site-specific strategies developed to maintain a systematic approach despite variation in search functionality across sources. Records retrieved from these sources were screened independently by two authors (CB/LZ) at the title and abstract stage. Where searches returned very large numbers of records, the first 100 results were screened to ensure feasibility while maintaining consistency across sources. Potentially relevant records were then assessed at full text by two authors (CB/LZ). Discrepancies were resolved through discussion between the two reviewers until consensus was reached; a third reviewer was therefore not required.

Data from peer-reviewed and grey literature sources were extracted into Microsoft Excel by two authors (CB/LZ). To ensure accuracy and reduce the risk of bias, both authors first extracted data from a subset of peer-reviewed articles (*n* = 7). Discrepancies were resolved through discussion. Extracted data included bibliographic information comprising the title, authors, and year of publication; study setting including city and country; study design including study type and method of data collection; participant characteristics including sample size, age, gender, and underserved group characteristics; the type of healthcare data addressed; analyses conducted; and main findings (S1 Table). A data extraction framework for the main findings was developed, informed by previous literature and in consultation with senior researchers with qualitative expertise (AMB/JA), to capture CYP and underserved groups’ perspectives, attitudes, and concerns comprehensively.

### Quality assessment

Study quality was appraised using the Mixed Methods Appraisal Tool (MMAT) [49]. This tool has been widely applied in mixed-methods systematic reviews and can be used to assess the quality of qualitative, quantitative, and mixed-methods studies. Two authors (CB/LZ) independently appraised a subset of articles (*n* = 5) to ensure consistency and reduce the risk of bias. The remaining articles (*n* = 12) were then assessed by either CB or LZ, with regular discussions to resolve queries. No studies were excluded on the basis of quality assessment scores.

### Data synthesis

Data were synthesised narratively using the JBI convergent integrated approach [50]. Quantitative findings were transformed into textual descriptions through “qualitisation” to enable integration with qualitative data, and all data were analysed together. Two authors (CB/LZ) reviewed the extracted data and applied a coding framework informed by the aims of the review, namely underrepresented groups’ perceptions, attitudes, and concerns regarding the use of healthcare data for research. Data were deductively coded and organised into descriptive themes. Emerging themes were discussed regularly with senior researchers with qualitative expertise (AMB/JA) to ensure that they accurately reflected the data. This was an iterative and reflective process, with themes refined through ongoing discussion and consideration of how researchers’ positionalities and perspectives might shape interpretation.

## Results

The database search identified 10,989 records. After importation into Covidence and deduplication, 6,778 records were retained. Following title and abstract screening, 129 potentially eligible articles underwent full-text review. Of these, 115 were excluded, leaving 14 studies for inclusion. A further three articles were identified through bibliography searches (*n* = 2) and online repository searches (*n* = 1). The study selection process is shown in the PRISMA flowchart (Fig 1).

**Fig 1:**
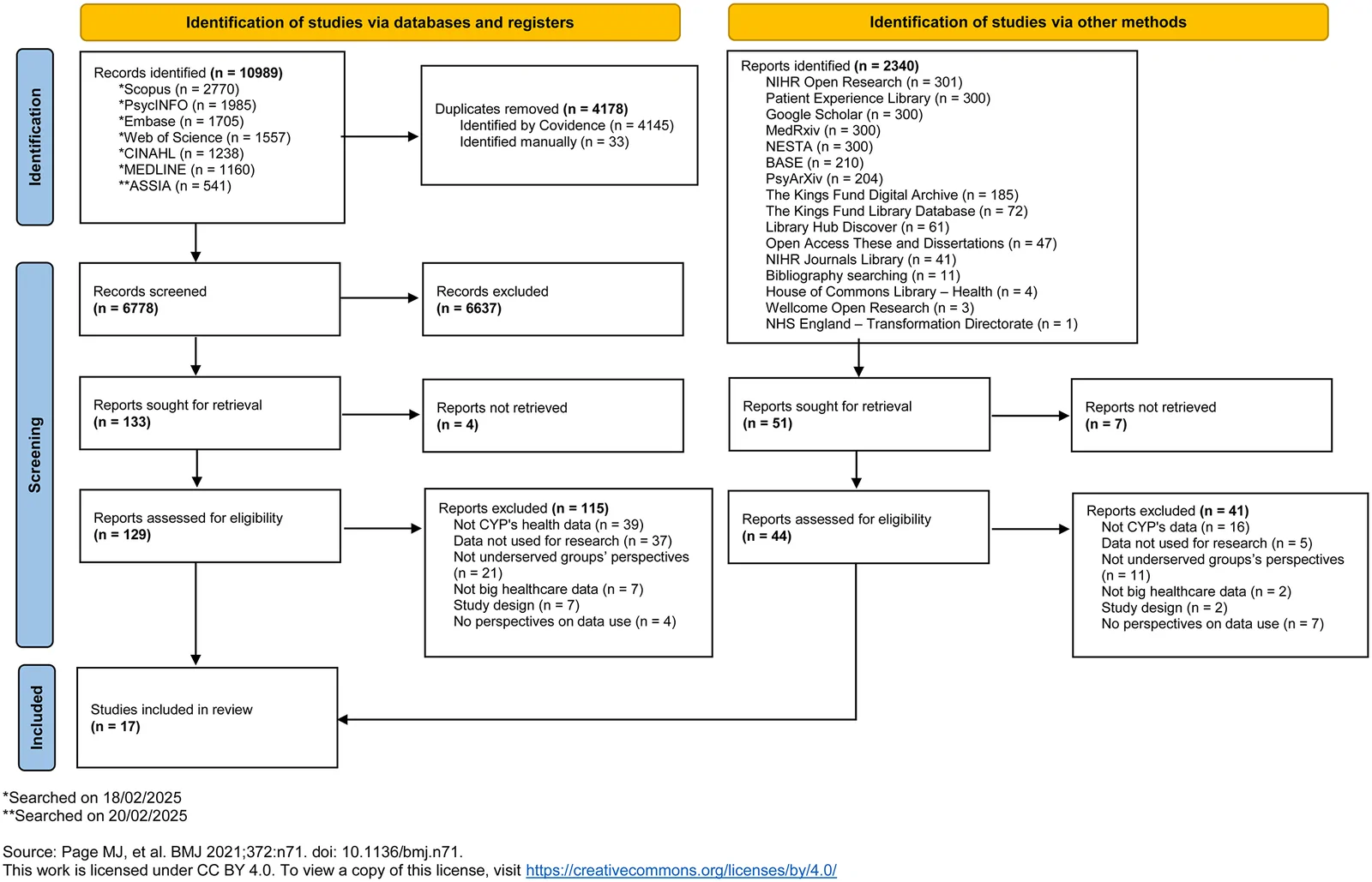
PRISMA flow diagram.

### Study characteristics

Table 2 summarises the characteristics of the included studies. Most were conducted in the UK (*n* = 7), followed by the US (n = 4) and Canada (n = 2), with one study conducted in each of Italy, Mali, New Zealand, and Norway. The perspectives of CYP (*n* = 10) and parents, carers, and/or guardians (*n* = 6) were represented, with one study reporting the perspectives of both groups. Seven studies represented the perspectives of CYP specifically, while others examined underserved groups including CYP with physical health problems (n = 6), ethnic minority groups (n = 1), mental health service users (n = 1), CYP with neurodevelopmental conditions (n = 1), and sexual and gender minority CYP (n = 1). Across studies, 4,710 CYP and 1,443 parents, carers, and/or guardians contributed perspectives, with one study (*n* = 108) integrating the experiences of both groups. Six studies used qualitative study designs, six used quantitative study designs, and five used mixed-methods approaches. Most studies (*n = 15*) were published in peer-reviewed journals, with two identified through grey literature sources.

**Table 2:** Study characteristics.

Underserved group	Study reference	Study aim	Setting and sampling	Perspective	Sample (N, gender, age)	Type of healthcare data	Study design and data collection method
**CYP**	Audrey et al., (2016)	Examining participants’ perceptions of data linkage, their views on its advantages and disadvantages and a detailed exploration of any apprehensions about data linkage processes. [...] In this paper we focus specifically on the issue of consent.	Bristol, UK Random and purposive sampling	YP (aged 17–19 years)	YP (N = 55; 56% female; aged 17–19 yrs)	Electronic administrative data	Qualitative exploratory study design Interviews
	Carey et al., (2024)	We aimed to determine the preferences of UK youth for a future global data bank of mental health data and capture their views on the collection, storage, and sharing of mental health data.	UK Purposive sampling	YP (aged 16–24 years)	YP (N = 46; 78% female; median age 19 yrs)	Mental health data	Qualitative exploratory study design Deliberative democracy session
	Helms et al., (2013)	(1) To assess young people’s [...] understanding of current systems of pharmacovigilance and pharmacoepidemiology, (2) To establish their knowledge of the uses of health data for research and planning, and (3) To identify any concerns and how these could be addressed.	Scotland, UK Convenience sampling and snowballing	YP (aged 14–16 years)	YP (N = 10 in focus groups and N = 132 questionnaire; 64.7% and 45.5% female respectively; aged 14–16 yrs)	NHS administrative data	Mixed methods study design Focus groups and questionnaire
	Kong et al., (2015)	We [...] developed a survey to explore the opinion of adolescents [...] regarding biobank participation and related subjects. In particular, we addressed willingness to donate biospecimens, appropriate age of assent and the perceived importance of reconsent when they reach the age of majority.	British Columbia, Canada Purposive sampling	YP (aged 14–18 years)	YP (N = 227; 52% female; M age 15.7 yrs)	Genetic data	Quantitative exploratory study design Surveys
	Sieberts et al., (2023)	The aims of the quantitative study arm were to understand (1) preferences of study participants regarding governance of their data and how it is accessed and (2) if governance policies affect study enrolment. The aims of the qualitative study arm were (1) to identify the consensus data governance model(s) for a [...] global mental health databank [...] and (2) to understand the concerns, hopes, and expectations of pan-national youth for such a databank.	UK Opportunity sampling	YP (aged 16–24 years)	YP (N = 3575 in the quantitative and N = 143 in qualitative study; 74.6% female in the quantitative study and not specified in qualitative study; 16–24 yrs)	Mental health data	Mixed methods study design Mobile-app mediated survey and deliberative democracy
	Shandhi et al., (2024)	In this study, we aimed to characterize the ownership and usage of smart devices among patients from an expansive academic health system in the southeastern US and understand their willingness to share data collected by the smart devices.	North Carolina, US Purposive sampling	YP (aged 18–25 years) part of a patient advisory group	YP (N = 24; 70.8% female; 18–25 yrs)	Health data collected by smart devices	Quantitative cross-sectional study design Survey
	Wild et al., (2023)	The aim of this study was to understand the views and expectations of children, young people [...] with regards to child health data storage, linkage and consent for data use in NZ.	New Zealand Convenience sampling	CYP (aged 5–16 years)	CYP (N = 14; 40% female; aged 5–16 yrs)	Health data	Qualitative exploratory design Semi-structured focus groups
**Ethnic Minorities**	Halverson & Ross (2012)	Our project focuses on whether the attitudes and values of an informed public cohere with these guidelines. More specifically, we focused on whether the guidelines resonated with the attitudes and values of informed African-Americans parents from diverse socioeconomic communities.	Chicago, US Convenience sampling	African American parents	Parents (N = 45; 76% female; M age 41 yrs)	Genetic data	Mixed methods study design Deliberative engagement with pre- and post- surveys
**Mental Health Service Users**	Koposov et al., (2024)	The present study aimed to 1) access mental health service users’ opinions about collaboration between municipal services and the specialist health service; 2) investigate service users’ awareness of health authorities sharing EHRs information; and 3) identify service users’ expectations towards the Health Platform.	Norway Purposive sampling	CYP and parents/ guardians	CYP mental health service users and parents/ guardians (N = 108; 72% female; 39% aged 35–44 yrs).	Electronic health records	Quantitative cross-sectional study design Questionnaire
**Neuro-developmental Conditions**	Raspa et al., (2020)	The purpose of this study was to examine parental preferences for researchers accessing their child’s electronic health record across 3 groups: those with a child with (1) a known genetic condition (fragile X syndrome, FXS), (2) a suspected genetic condition (autism spectrum disorder, ASD), and (3) no known genetic condition (typically developing).	US Convenience sampling	Parents of children (aged 14–17 years) with FXS or ASD	Parents (N = 1,008; 91.1% female; average age of child = 20.2 yrs)	Electronic health records	Quantitative non-randomised control study design Discrete Choice Experiment (DCE)
**Physical Health Conditions**	Chow et al., (2024)	We aimed to conduct a Canada and US-focused mixed methods study involving caregivers of children aged <18 years and youth aged 16–17 years to understand the relationship between socio-demographic characteristics [...] and “trust in” and “willingness to use” a digital platform to store and share personal health information (PHI) for clinical care and research.	Canada Purposive sampling	Caregivers of a child with a chronic disease and youth	Respondents (N = 198 caregivers of a child with a chronic disease and N = 173 youth; unspecified; unspecified)	Personal health information	Mixed methods study design Survey and discussion
	Hughes et al., (2023)	We invited CYP and their carers to a patient and public involvement (PPI) workshop to learn how their healthcare data is used and to: 1) Identify current barriers to trust regarding the use of healthcare data for research; 2) Determine ways to increase public and patient; confidence in the use of healthcare data in research 3) Define areas of research importance to CYP and their carers using healthcare data.	UK Purposive sampling	YP (aged 16–25 years) diagnosed with cancer before aged 20 yrs and carers	YP and carers (N = 14 and N = 10, respectively; 71.4% and 80% female, respectively; 58.3% aged 16–25 yrs)	Healthcare data	Qualitative PPIE study design Workshops
	Salvaterra et al., (2015)	The study aims to expand the current knowledge on this topic by determining whether the nature of the illness, its seriousness and the broader health conditions of the patient may influence parental decision-making about the participation of their children in biobanks.	Italy Purposive sampling	Parents of children with acquired or inherited genetic neurological conditions (i.e., Encephalitis, traumatic brain injuries, Duchenne dystrophy, or Williams syndrome)	Parents (N = 30; 8 couples and 14 individual parents; or specified; children aged between pre-school and adolescence)	Genetic data	Qualitative exploratory study design Focus groups
		Scollon et al., (2014)	The goals of this manuscript are [...] to explore (1) the proportion of parents who decline enrolment of their child on this clinical WES study and their reasons for doing so, (2) a comparison of clinical and demographic factors [...] between patients whose parents agree to or decline study enrolment, and (3) the preferences of parents for participation in optional genomics study procedures such as inclusion of recessive carrier data in WES reports and deposition of patient data into research databases.	Texas, US Purposive sampling	Parents of CYP with cancer	Patients (N = 100; 45% female; median age 5.1 yrs)	Genetic data	Quantitative cross-sectional study design Questionnaire
		Traore et al., (2015)	This research aimed to examine the range and diversity of experiences and understandings of recruitment processes for a MalariaGEN study.	Mali, West Africa Purposive and snowballing sampling	Parents of children with severe malaria	Parents (N = 52 parents of children with malaria; 40% female; not specified)	Genetic data	Qualitative rapid ethical assessment study design Focus groups
		West & Cumella (2018)	Asthma UK conducted a survey designed to gain insight into people with asthma’s attitudes to the sharing of personal health data for research and service improvement, alongside their views on the use of new technology and data in their asthma care.	UK Not specified	YP (aged 17 yrs or under) with Asthma	YP (N = 100; 45% female; 100% aged 17 yrs or under)	Personal health data	Cross-sectional quantitative study design Survey
**Sexual and Gender Minorities**		Matson et al., (2019)	This paper explored SGM youth’s perspectives on sharing de-identified survey responses from sexual health studies and blood samples from HIV testing studies and youth’s recommendations to help protect adolescent participants when these data are shared with other researchers.	UK Purposive sampling	YP (aged 14–17 years)	YP (N = 197; 60.4% female at birth; M age 16.5 yrs)	Blood samples	Mixed methods cross-sectional study design Questionnaire

### Quality appraisal

The quality of the included studies ranged from low to very high. One study was rated low quality, meeting two criteria. Three studies were rated moderate quality, meeting three criteria. Four were rated as high quality, meeting four criteria. Six met all five criteria and were rated very high quality. Qualitative studies were generally rated more highly. The three studies identified through grey literature searches were not appraised, given the nature of grey literature reports. Quality assessment scores for the included studies are provided in the supplementary materials (S2 Table).

### Narrative synthesis

Findings are presented in four sections: conditional support for the use of big data in CYP healthcare research; perceived benefits of using big data in paediatric research; concerns about data-driven approaches to CYP healthcare research; and conditions required to ensure that data-driven research approaches are acceptable and equitable.

### Conditional support for the use of big data in CYP healthcare research

Across the included studies, underrepresented groups expressed broadly supportive views towards the use of big data in CYP healthcare research, but this support was conditional. Participants’ willingness to share data was shaped by an ongoing evaluation of perceived benefits, trust in institutions, data sensitivity, and the extent to which they retained control over how data are used. Findings across these interrelated dimensions of conditionality show how support, concern, and conditions for acceptability were part of a single evaluative process rather than distinct domains.

#### Patterns of willingness.

Across several types of data—including EHRs [51–54], genetic data and biological samples [55–60], healthcare data including data collected through digital or smart devices [61–65], and mental health data [66,67]—participants reported generally high levels of willingness to support big data use in CYP healthcare research.

High levels of willingness were observed in contexts where the purpose of data use was clearly linked to health benefit and where the institutional context was perceived as legitimate. In a study involving youth mental health service users and their parents, carers, or guardians, 81% reported willingness to share health data with trusted health authorities, and 83% considered it ethical to share health records for research [51]. Similarly, among caregivers, 88.9% reported willingness to share their own or their child’s personal health information if it contributed to progress in non-profit health research, compared with 72.9% of youth in the same study [65]. Consistent with these findings, a grey literature report found that 89.4% of young people believed that the NHS should have unrestricted access to linked anonymised healthcare data for research purposes [54]. Another grey literature study reported that 83.8% of young people were comfortable sharing their data to support the development of new asthma treatments, and 95.9% were comfortable sharing their data to develop tools to identify individuals at risk of an asthma attack [64]. High levels of willingness may not simply indicate generalised support, but reflect contexts in which the public benefit is legible, the health relevance is clear, and the institutional context feels legitimate.

Willingness was particularly high in clinical contexts, where potential benefits were direct and tangible. In one study, 96.6% of parents reported willingness to donate their child’s data to a biobank for research [57]. Similarly, among parents of children with cancer, 94–95% consented to the use of diagnostic tumour samples and the collection of additional tumour or blood samples for research, 90% consented to the sharing of de-identified genetic and clinical data with other researchers, and 87% consented to the deposition of such data in a research database [58]. However, willingness to share de-identified biological samples was lower among sexual and gender minority youth, of whom 68% reported willingness to share blood samples with other researchers [60]. Although willingness may be higher in clinical contexts where potential benefits are direct and tangible, it may be lower among groups for whom data sharing carries additional social or identity-related risks.

Willingness was not uniform and often became more conditional where data were perceived as more sensitive or less clearly linked to health benefit. Several studies [53,55,56,62,63] reported mixed or conditional willingness, rather than unequivocal support. In one study of smart device data, 67% of young people reported willingness to participate in future studies involving the sharing of activity data, whereas 29% responded “*maybe*” and 4% responded “*no*”, indicating substantial uncertainty [63]. Young people also reported mixed views regarding data linkage [62]. Although most supported linkage of some data types, such as teenage pregnancy and state benefits (34/55) or birthweight and future health outcomes (36/55), views were more divided in relation to linkage involving mental health and criminal records (20/55) [53]. Willingness may be shaped by perceived sensitivity and type of data, with greater hesitation where data are more personal, potentially stigmatising, or less clearly linked to health benefit.

#### Conditions shaping willingness and acceptability.

Participant characteristics appeared to influence willingness in studies comparing the perspectives of young people and parents [54,55,63]. For example, young people were more likely than parents to support continued use of samples and were less likely to refuse DNA donation [55]. Differences by ethnicity were observed in one study, in which Black participants showed lower willingness to share blood samples and Asian participants showed higher willingness [60]. However, two other studies reported no significant differences in willingness according to age, ethnicity, or gender [58,67]. While demographic differences may shape willingness in some contexts, the evidence remains limited and inconsistent, indicating that broader contextual and relational factors may be more influential than individual characteristics alone.

Research context and lived experience also appeared to shape willingness. One study found that 52% of adolescents approached in clinical settings were willing to donate DNA, compared with 47% of adolescents approached in school-based settings [55]. Participants approached in clinical settings were also consistently more willing to donate DNA across scenarios involving additional procedures, additional blood samples, or leftover samples [55]. By contrast, one other study found no significant differences in willingness according to lived experience [67]. Healthcare service use and lived experience of illness may increase perceived relevance and legitimacy of research, although this is not uniform across all contexts.

Trust in institutions and perceptions of acceptable uses of sensitive data also influenced willingness. One study found that participants were more willing to support research conducted by public, academic, or non-profit institutions or by individuals such as healthcare professionals whose role was perceived to involve advocacy for CYP health [66]. Trust was also shaped by perceptions of appropriate data use. Participants in India and South Africa expressed a preference for their mental health data to be used only for mental health research, whereas participants in the UK expressed a slight preference for their data to be used for other forms of healthcare research [67]. Perceived data sensitivity further influenced willingness, with participants expressing a preference for research using non-identifiable or less sensitive information [52]. Willingness is closely tied to perceptions of trust, legitimacy, and appropriate use, with participants favouring contexts in which data use aligns with expectations of care, relevance, and ethical oversight. These findings suggest that willingness is not a fixed attitude, but a context-dependent judgement shaped by how participants weigh perceived benefit, trust in institutions, data sensitivity, and the degree of control they retain over data use.

### Dimensions of conditional support

#### Perceived benefits and motivations.

Societal and public health benefit emerged as a central justification for the use of CYP healthcare data in research. CYP described participation as a way to “*give back*” [55] and as “*a good thing to do*” [57]. Perceived legitimacy was closely linked to clarity of purpose. Research on heart disease and asthma was viewed as having clearer societal value than studies using pregnancy or mental health data, whereas linking birthweight to later health outcomes was seen as serving the public good [53]. Sexual and gender minority youth also expressed altruistic motivations; in one study, helping the community was cited as a reason for limited concern about data sharing [60]. In mental health contexts, use of big data was perceived as a way to reduce stigma and improve public understanding. Young people felt that findings from big data research could help normalise mental health difficulties, reduce stigma, and encourage broader discussion [66,67]. A grey literature source similarly reported that data linkage was perceived to benefit wider society by 74% of young people and 86% of parents or guardians [54].

Participants also perceived data sharing as contributing to research and scientific progress, which was typically understood as part of a broader collective benefit, particularly where it was linked to improved health outcomes. Several studies reported that participants were motivated by a desire to support research and enable new discoveries. Young people described data sharing as a way of supporting mental health research and wanted data to be widely accessible to facilitate *“research, creativity and discovery”* [66]. Similarly, 69.1% of sexual and gender minority youth reported that sharing blood samples with researchers would be helpful [60]. Parents of CYP with physical health conditions described participation as a means of supporting research development [57] and advancing research into rare cancers [61], with 80.0% of parents of children with newly diagnosed cancers agreeing to study enrolment [58]. Strong support for research was also evident among caregivers more broadly, with 89.0% of youth and 97.0% of caregivers indicating that an integrated platform would be helpful to researchers [65].

Big data sharing was also viewed as a means of helping others with similar lived experience. Across several underrepresented groups, CYP expressed willingness to support initiatives that might benefit sick children [55] or children with similar health conditions [62]. This motivation was particularly evident among mental health service users, 84% of whom perceived value in sharing health information to help others with similar diagnoses [51]. Sexual and gender minority youth reported similarly strong peer-oriented motivations, with 98.5% stating that it felt good to help other LGBTQ teens by taking part in sexual health research [60]. Parents of children with Fragile X Syndrome (FXS) or Autism Spectrum Disorder (ASD) also expressed stronger preferences for studies on personally important topics than parents of typically developing children [52]. Motivations often reflected a relational and community-based form of altruism, grounded in solidarity with others who share similar diagnoses, identities, or experiences of marginalisation. This form of benefit may help explain why some groups express support for data use, as participation is understood as contributing to the wellbeing of their own communities, rather than to science in the abstract.

In contrast to these collective motivations, participants also identified more immediate and tangible personal benefits of using big data for CYP healthcare research. Parents of CYP with physical health conditions described participation as an opportunity to access high-quality care, support appropriate diagnosis and treatment, and monitor their child’s health status [59]. Consent discussions were, in some cases, experienced as empowering, enabling families to take a more active role in health management [62]. A grey literature source similarly found that 72% of young people and 91% of parents or guardians perceived individual benefit from data linkage [54]. The return of results was especially valued across several groups. Ethnic minority parents expressed particularly high interest in receiving individual results relating to their child (97%), with less interest in aggregated findings [56]. Parents of children with FXS or ASD also showed stronger preferences for studies returning summary or individual results than parents of typically developing children [52]. Alongside collective and community-oriented motivations, personal or family-level benefit plays an important role in shaping willingness, particularly where benefits are direct, tangible, and relevant to the child’s care. However, these perceived benefits were rarely sufficient on their own. Rather, they formed one component of a broader evaluative process in which participants also considered risks, trustworthiness, and the conditions under which data would be used.

#### Concerns and privacy risks.

Privacy, confidentiality and the risk of disclosure were prominent concerns across the included studies. Participants expressed particular unease about digital platforms and electronic data handling. In one study, 91.4% of caregivers and 92.5% of youth reported being very or somewhat concerned about web-based data privacy and security, especially in relation to the electronic storage and sharing of personal health information [65]. Similar concerns were reported by parents of CYP with acquired or inherited genetic neurological conditions, whose unease centred on the risk of privacy breaches involving genetic data [57]. By contrast, privacy concerns were less frequently raised by parents of CYP with cancer, among whom only a small minority (3%) identified privacy as a key issue [58].

Privacy concerns were closely tied to doubts about the adequacy of anonymisation. Young people showed awareness that anonymised data might still be re-identified, particularly when datasets were linked or when research involved small or distinctive populations [53,66]. Some questioned whether anonymisation could reliably prevent individuals from being singled out, especially where data were reused or combined across studies [66,67]. These concerns were heightened in relation to biological samples, such as blood, which were perceived as inherently identifiable because of their genetic content [60].

Concerns about privacy were also shaped by the possibility of stigma and unwanted disclosure. Young people worried that the use of sensitive data, including mental health, sexual health, or socioeconomic information, might reinforce negative stereotypes or further stigmatise already marginalised groups [53,62]. Sexual and gender minority young people raised specific concerns about the disclosure of identifiable information, including fears that research data use could reveal sexual or gender identity, HIV testing status, or sexual activity to family members or others without consent [60].

Participants also raised concerns about the long-term implications of data storage and reuse, particularly where data were collected in childhood. Caregivers worried that information shared early in life might later be linked back to their child and affect future opportunities, including employment or access to insurance [65]. Young people similarly expressed unease about long-term storage of biological samples and uncertainty about how their data might be used in future, including for purposes not anticipated at the time of collection [60].

Participants were not naïve about anonymisation, but instead understood it as partial, contingent, and context-dependent. These concerns may not be resolved by the use of anonymisation alone, particularly where datasets were linked or involved small or identifiable populations, suggesting that technical assurances alone may be insufficient in fully addressing perceived privacy risks.

A second major area of concern related to data misuse, trust, and commercial exploitation. Participants frequently raised concerns about the profit-driven use of CYP health data, particularly by private or commercial organisations. Young people expressed discomfort about sharing their data with private companies, especially where health data were perceived to be used for financial gain rather than direct health benefit [54,66]. Participants worried that data might be sold to third parties or reused for profit, and some expressed distrust towards advertising, insurance, and pharmaceutical companies as data handlers [65,66]. These concerns reflected apprehension about commercial motives rather than blanket opposition to research use.

Concerns also extended to harmful or unethical applications of data. In some studies, participants raised fears that researchers might have questionable intentions or that research might contribute to harmful outcomes, including eugenic practices [66]. Caregivers of CYP with genetic conditions expressed concern that biological samples could be used for illegal purposes, such as the development of biological weapons [57]. Others worried about how data might be misused in countries where mental health stigma is high or where there are histories of poor human rights protections, raising the possibility that data could be used in discriminatory ways [66].

Participants also expressed concern about malicious or unauthorised access, including by hackers or by individuals acting in bad faith within otherwise reputable research institutions [67]. Young people described fears of sabotage, corruption, or intentional destruction of data, and of the possibility that such actions could further disadvantage marginalised groups [66]. These concerns related both to external security threats and to internal failures within trusted systems.

Mistrust regarding data misuse was often grounded in historical and community-level experience. Participants referred to previous examples of data misuse or security breaches as reasons for distrusting researchers, institutions, and data handling procedures [53,62,67]. In some settings, rumours that blood samples collected during studies had later been sold reinforced scepticism towards researchers and healthcare professionals [59]. Media reporting also contributed to mistrust, particularly where coverage focused on patient data being sold to private companies or used for non-research purposes [61]. Receiving unsolicited advertising after data sharing further reduced confidence and blurred the distinction between data use for research and data use for marketing [61].

Concerns about misuse included fears of misrepresentation and bias in how data are analysed and interpreted. Participants worried that inaccurate or recreated data could distort research findings and have real-world consequences, particularly for underserved groups [66]. Participants also expressed concerns that, in large datasets, indigenous ethnic minorities may be overlooked, which could reinforce existing inequalities through data-driven research [62].

These commercial concerns suggest that participants may not be opposed to big data use in research per se, but may evaluate it in relation to the perceived purpose, benefit, and accountability of those accessing and using data. Participants appeared to distinguish between actors and uses, expressing greater acceptance of data use aligned with public or health benefit, and greater concern where data use appeared profit-driven, unaccountable, or detached from care. Concerns about commercial use may reflect moral distinctions rather than simple opposition to research.

A third area of concern related to data governance, transparency, and loss of control. One study identified concerns about data access arrangements, with participants expressing mixed views regarding databank ownership and access by different actors [67]. Some young people raised concerns that databanks owned by private companies could enable data misuse for commercial gain, whereas others acknowledged that private ownership might help sustain databanks financially [67]. Participants also questioned who should be permitted to access data, including whether unrestricted access, or access by researchers from particular countries or institutions, was appropriate. For some, centralised storage of data in a single location increased perceived risks of misuse and loss of control [67].

Concerns about governance also extended to commercial involvement and conditions of access. Many participants preferred that their data should not be used to generate profit, and felt that commercial organisations should pay for access where such use occurred [67]. Views on data access contracts were ambivalent. Some participants worried that contracts could be violated or forged, whereas others regarded them as an important mechanism for accountability and agreed use [67]. These perspectives reflected uncertainty about whether formal governance mechanisms could reliably protect participants’ interests.

These concerns were closely linked to a desire for transparency and awareness. Young people expressed discomfort with the possibility that researchers might use their sensitive data without their knowledge, or that data held within biobanks could become inaccessible to them over time [53,62]. School-aged adolescents, in particular, reported unease about not knowing which studies their donated data or samples would be used for, highlighting the gap between initial data collection and ongoing communication about subsequent use [55].

Questions about consent and decision-making authority further shaped concerns about control. Participants expressed uncertainty about whether consent was required for the use of data or samples from individuals who had died [53]. Some parents felt uncomfortable making enrolment decisions on behalf of their child and believed that CYP should be involved in decision-making where possible [56]. Parents were often described as acting as gatekeepers between researchers and their child [56], a role not only associated with responsibility but also with uncertainty about the longer-term implications of those decisions.

Governance concerns also intersected with questions of fairness and equity. Participants worried that data access fees, storage costs, or technical requirements could prevent smaller research teams, or those with limited funding, from accessing data, thereby shaping which research questions were pursued [66,67]. At the same time, some participants viewed access fees as a potential safeguard, either as a barrier to misuse or as evidence of researcher commitment [67]. Broader structural barriers, including the absence of global governance frameworks, language barriers, currency conversion, and differences in identification systems across countries, were also identified as factors that could restrict equitable access and oversight [66]. Young people and caregivers further expressed concern that individual data points stored in large datasets might fail to represent a whole person or their circumstances adequately, raising questions about whether data governance frameworks can account for context, nuance, and lived experience [62].

Concerns about governance may reflect broader concerns about control: whether data use remained transparent, accountable, and subject to meaningful oversight over time. Risks may not only be understood in terms of privacy or trust, but in relation to a broader sense of losing control over who accesses data, how it is used, and how decisions are made across the data lifecycle.

A fourth theme concerned physical and emotional barriers to data sharing. Concerns about physical discomfort and embodied distress were commonly reported by adolescents and caregivers. Adolescents described fears of needles and medical procedures, particularly where participation involved additional blood samples beyond routine care [55,60]. Some reported a more general unease about blood donation or about the idea of their blood samples being shared, and a small number indicated that discomfort with blood draws would deter participation [60]. Caregivers similarly raised concerns about causing physical pain or distress to their child during specimen collection, particularly in relation to genetic data [57].

Participants also described emotional burden linked to the timing and context of research requests. Among parents of CYP with cancer, the most common reason for declining participation (10%) was feeling overwhelmed by their child’s recent diagnosis and not wishing to engage in additional research at that point [58]. A smaller proportion (2.5%) reported anxiety about receiving genetic testing results, suggesting concern about emotional readiness rather than opposition to data sharing itself [58]. Similarly, some parents expressed unease about testing data for multiple conditions, particularly where there was a possibility of identifying stigmatised or unexpected diagnoses, such as HIV [59].

Barriers to participation were also shaped by the perceived absence of direct or personal benefit. Parents of children with FXS or ASD expressed lower preferences for studies that did not return individual results than parents of typically developing children, which reduced willingness to participate in data sharing [52]. In one qualitative account, a parent described perceiving limited value in biobank participation because their child’s condition was attributed to a traumatic event, which reduced expectations of benefit from research involvement [57]. Across studies, limited perceived individual benefit reduced motivation to engage, particularly where participation involved additional effort or discomfort. These concerns were reinforced by the practical and cumulative burdens of participation, with a small proportion of parents (2%) reporting that they did not want any further blood to be taken for research purposes [58]. These concerns did not necessarily translate into outright opposition, but instead informed the conditions under which data use was considered acceptable.

#### Governance, control and consent.

Building on these concerns about control, maintaining control over personal data emerged as a key condition for acceptable data sharing across underserved groups. Young people repeatedly emphasised the importance of retaining ownership over their data and argued that sensitive information should not be accessible to everyone [53,54,66]. Multiple studies found that young people wanted involvement in consent procedures [54,55,60], and two studies reported that adolescents felt ready to contribute to medical decision-making at around 14 years of age [54,55]. Although some younger adolescents indicated that patient assent was unnecessary, or that they were content for parents to make decisions on their behalf [55,61], participants generally felt that, as children mature, they should increasingly make such decisions for themselves as they move from providing assent to consent. Across age groups, CYP identified multiple stakeholders, including themselves, parents, and healthcare professionals, as appropriate data controllers [61,62]. Parents across different underserved groups, including ethnic minority families and parents of children with neurological conditions, also expressed a desire to retain control over their child’s health data and remain involved in decision-making processes [56,57]. Robust ethical safeguards and scrutiny were also identified as essential conditions.

Across these studies, consent was understood as a developmental and relational process, rather than a fixed or one-time decision. Decision-making authority was seen to evolve over time, shaped by age, experience, and context, while responsibility was shared across children, families, and professionals. This highlights a temporal challenge for data governance in paediatric contexts: decision-making capacity changes as children mature, whereas data may be stored and reused over extended periods. As such, participants’ views point towards the importance of ongoing involvement, revisitable consent processes, and governance approaches that can accommodate changing roles and responsibilities over time.

Young people also emphasised the importance of strong ethical procedures, with privacy, anonymity, and confidentiality regarded as core safeguards requiring appropriate training for those handling data [66]. Young people also indicated that research carrying greater potential for harm should be subject to greater scrutiny than lower-risk projects [66,67]. In addition, access to health data by non-NHS organisations was viewed by some as something that should be chargeable [54].

Security and governance were also central to participants’ conditions for acceptability. Concerns about appropriate storage and governance structures were common across studies. Young people expressed mixed views about optimal storage arrangements, although secure servers were generally viewed as more acceptable than downloading data [62,67]. In one study, governance preferences varied by geographical context: young people in India and South Africa favoured a professional review panel to control data access, whereas those in the UK preferred a democracy model, followed by a professional review panel, with community and volunteer panels also receiving some support across sites [67]. Multiple studies indicated that young people and parents felt that health organisations such as the NHS in the UK or government bodies, rather than private companies, should manage health data [54,66,67].

Anonymisation was repeatedly identified as a critical condition for acceptable data use, as it was consistently viewed as important for protecting privacy and confidentiality [53,54,61]. Parents of CYP with neurodevelopmental conditions preferred studies using non-identifiable rather than identifiable information, and non-sensitive rather than sensitive information [52]. Several studies also demonstrated support for removing identifiable information to protect confidentiality while still allowing donor identification where necessary [57,60]. Mechanisms such as restricted access, transparency, and ongoing involvement in decision-making were therefore valued as ways of maintaining legitimate social control, and in turn enabling conditional support for data use.

## Discussion

This review is the first to systematically examine the perspectives of underrepresented groups on the use of big data in CYP healthcare research. The included studies captured the views of CYP and underserved groups, including those with physical health conditions, ethnically minoritised backgrounds, experience of mental health service use, neurodevelopmental conditions, and sexual and gender minority identities. Perspectives were reported directly by CYP, and by parents, carers, or guardians acting as proxy decision-makers in relation to the use of CYP healthcare data. Across studies, underrepresented groups expressed broadly favourable, but conditional, support for the use of big data in paediatric healthcare research. Several studies suggested that willingness to share CYP health data was shaped by participant characteristics, research context and lived experience, trust in institutions, and perceptions of acceptable uses of sensitive data, particularly where commercial involvement or highly sensitive information was involved. Underserved groups may not fundamentally oppose data-intensive research, but instead evaluate it through a more acute sensitivity to trust, misuse, and institutional accountability, reflecting the fact that these risks are not hypothetical within their lived experience.

This conditionality may be understood as a form of context-sensitive ethical reasoning, in which participants actively evaluate the legitimacy, risks, and purposes of data use. Perceived benefits clustered around broadly altruistic themes, including societal and public health benefit, supporting research and scientific progress, helping others with similar lived experience, and perceived healthcare benefit including the return of individual results. Alongside these perceived benefits, underrepresented groups also raised consistent concerns about data-driven paediatric research. These centred on privacy, risk of re-identification, long-term data storage, data misuse, particularly commercial exploitation, limited transparency, and loss of control over data governance. Across studies, participants also articulated clear conditions under which the use of big data would be considered acceptable. These included robust anonymisation procedures, independent ethical oversight, and developmentally appropriate consent processes. These findings suggest that underrepresented groups evaluate big data use in CYP research through a contextually grounded ethical lens.

These findings are broadly consistent with previous work on public attitudes towards the use of health data for research, which has similarly identified conditional support for data-driven health research [7–9,11,12]. Existing studies suggest that individuals are generally willing to share health data when the purpose is clearly defined, socially beneficial, and governed by robust safeguards. Concerns about privacy, re-identification, and potential commercial misuse are also widely reported across studies examining attitudes towards digital health data use [7–11,68]. The present findings extend this literature by demonstrating how such conditions are shaped by experiences of marginalisation, proxy decision-making, stigma, and differential exposure to institutional harm. The emphasis on transparency, robust governance, and the sensitivity of particular forms of health information, such as mental health or neurodevelopmental data, reflects the particular salience of these concerns for communities already subject to structural marginalisation within healthcare systems [18,21]. This is consistent with literature suggesting that historical inequities and prior experiences of exclusion shape how underserved communities evaluate research participation [18,20,21], and how they balance the risks and benefits of emerging data-driven approaches [34].

Although this review focused specifically on the use of healthcare data in research, related work on other forms of digital data points to similar tensions. The use of social media data for health research, for example, has been associated with societal benefit, but also with persistent concerns about privacy and misuse [69,70]. As the boundaries between health data and other forms of personal digital data become increasingly blurred, future research should examine whether underrepresented groups evaluate different forms of digital data sharing in different ways. In addition, the studies included in this review encompassed a broad range of data types, including EHRs, data linkage, biobanks, and digital or smart device data, which differ in their ethical and social implications. Participants’ responses may therefore reflect engagement with different research contexts rather than a single, unified understanding of “*big data*”, which may partly explain variation in participants’ views across studies.

Several important gaps in the current evidence base were apparent. First, despite growing recognition that underserved groups experience structural disadvantage in research participation [21], and their explicit prioritisation within frameworks such as NIHR-INCLUDE [17], relatively few studies have examined their views on the use of big data in CYP healthcare research. Among the included studies, only one explicitly explored the views of ethnic minority participants, with similarly limited representation of mental health service users, those with neurodevelopmental conditions, and sexual and gender minority youth. Populations frequently described as underserved therefore remain underrepresented even within research concerned with attitudes towards big data use in CYP healthcare contexts, and findings should be interpreted with appropriate caution.

Second, few studies examined attitudes towards cross-sector data linkage involving healthcare data, such as linkage between health records, education, and social care records. Linked administrative data are increasingly used to examine structural and institutional determinants of health and their influence on long-term outcomes [5,6]. Yet most included studies focused on health data or biobank-type models, suggesting the evidence base is lagging behind the real-world direction of data-driven research. The ethical and governance implications of cross-sector linkage remain insufficiently explored from the perspective of underrepresented groups.

Third, the perspectives of younger children, particularly those under 14 years, were rarely represented, limiting the extent to which findings could be meaningfully examined separately by age group. Six of the seventeen included studies focused on parental or caregiver perspectives acting as proxy decision-makers, which may not fully reflect CYP’s own views. Although this may reflect practical and ethical challenges in conducting research with younger participants [16], it limits understanding of how younger children conceptualise privacy, trust, and participation in research involving their health data. Consequently, the findings should be interpreted as representing the perspectives of both CYP and proxy decision-makers rather than CYP alone.

Finally, several studies suggested that concerns about privacy and confidentiality were less prominent in contexts involving acute or life-threatening illness. This may reflect implicit judgements about risk and benefit rather than an absence of concern, whereby the perceived benefits of research outweigh potential privacy risks when illness severity is high. These findings suggest that attitudes towards health data use are also shaped by the circumstances in which data-sharing decisions are made, including illness severity, perceived benefit, and prior experience of healthcare systems.

The findings have several implications for health research using big data. Research involving CYP health data must prioritise governance structures that are visible, intelligible, revisitable, and participatory. Acceptability appears closely tied to transparent governance, clear communication about who can access data and for what purposes, and visible safeguards against misuse or commercial exploitation. Concerns about opaque access arrangements, secondary use, and loss of control suggest that governance mechanisms must not only be robust, but must also be experienced as legitimate and communicated in accessible ways, including acknowledgment of future reuse, changing decision-making capacity, and the possibility of sensitive secondary uses. Child-friendly language may facilitate dialogue with CYP and their families [38]. Clear oversight structures and independent ethical scrutiny may therefore be essential for maintaining trust in large-scale digital health data initiatives involving underrepresented groups. Meaningful PPIEP throughout the design and implementation of governance processes may help address these concerns, particularly where mistrust is grounded in prior institutional experiences, and support more inclusive and responsive research practices.

The findings also highlight the need for consent models that recognise adolescents’ developing autonomy. Participants expressed a clear preference for age-appropriate consent processes that allow CYP to become progressively more involved in decisions about their data as they mature. Opportunities to revisit consent over time may be important for promoting a sense of agency, in keeping with recommendations by the ERIC project [38] and with children’s participation rights under Article 12 of the UNCRC [14,15]. Although informed consent that can be withdrawn at any time is widely recognised as good research practice [71], its practical utility has been questioned in the context of long-term biomedical research [72]. Similar concerns were reflected in participants’ views across the included studies, supporting the potential value of more flexible and responsive approaches [72]. Dynamic consent models may represent one possible approach to supporting CYP’s evolving capacities and expectations of ongoing involvement, transparency, and age-appropriate autonomy in longitudinal big data research.

### Strengths and limitations

This review has several strengths. To our knowledge, it is the first to synthesise evidence on the perceptions, attitudes, and concerns of underrepresented groups regarding the use of big data in CYP healthcare research. In line with best practice, the review was registered with PROSPERO and conducted in accordance with PRISMA guidance [39]. Quantitative, qualitative, and mixed-methods evidence was synthesised using the JBI convergent integrated approach [50], allowing integration across multiple forms of evidence. Inclusion of grey literature also reduced the risk of publication bias.

Several limitations should also be noted. Only studies reported in English were included due to language limitations within the authorship team, which may limit generalisability. This restriction may also have contributed to the predominance of studies conducted in Anglophone high-income countries (14 of 17 studies), particularly the UK, with limited representation of studies from low- and middle-income countries (LMICs) and the Global South. This is particularly important given the focus of the review on populations whose perspectives are already often underrepresented in healthcare and data-driven research.

Another significant barrier to generalisability stems from the limited number of studies conducted with underrepresented groups, meaning that these perspectives may be further marginalised in our review. For example, only one study each included ethnic minority perspectives, sexual and gender minority CYP, and mental health service users. The synthesis therefore potentially risks an overrepresentation of perspectives from CYP who might not experience the same barriers to healthcare research participation as their more marginalised peers. Any conclusions drawn from the synthesis should be interpreted with a contextual understanding of the structural differences that underrepresented groups experience when it comes to engaging with healthcare institutions and research conducted in such contexts.

Second, the use of qualitisation to integrate quantitative and qualitative findings introduces a potential risk of reporting bias [50], which may affect the validity of the synthesis. In addition, the included studies were heterogeneous in how “*big data*” was defined and operationalised, and in the methods used to assess attitudes, perceptions, and willingness to participate in research involving CYP health data, which raises the possibility of misclassification. These differences are not only methodological but also reflect variation in the types of data and research contexts being considered (e.g., EHRs, biobanks, digital device data), which may limit comparability across studies and influence how participants understood and perceived big data use in CYP research. Furthermore, given the limited evidence base and the heterogeneity of the included data contexts, it was not considered meaningful to synthesise perceptions according to specific data types. Consequently, findings should be interpreted cautiously, recognising that attitudes may vary according to the type of health data and research context considered.

Third, in line with MMAT guidance [49], no studies were excluded on the basis of methodological quality, as the MMAT discourages calculating overall appraisal scores. Nevertheless, nine included studies either did not meet one or more appraisal criteria or provided insufficient information for one or more criteria to be assessed. Consequently, findings from these studies should be interpreted with appropriate caution, as methodological limitations and insufficient reporting may reduce confidence in the reliability of some findings contributing to the synthesis.

Fourth, much of the evidence is derived from hypothetical willingness and attitudinal measures rather than observed behaviour in real-world data-sharing settings. Expressed comfort with sharing data therefore may not always translate to actual participation when decisions become concrete, particularly in contexts involving sensitive data or more complex governance arrangements. This is particularly salient considering most of the studies included in this review recruited populations who were already engaged in clinical or healthcare research processes, thereby potentially reflecting a preexisting willingness or favourability towards research participation. Therefore, it is important to contextualise findings on conditional support and willingness by considering the likely effect of selection bias stemming from such recruitment strategies.

### Recommendations for future research

There is a clear need for further research in this area. The evidence base remains particularly sparse in relation to the Global South, CYP living with comorbid disabilities, ethnically minoritised populations living in Global North countries, and CYP facing additional barriers to healthcare research participation because of intersecting marginalised identities. Further research should therefore prioritise the perspectives of underserved groups who remain underrepresented in studies of big data use in CYP healthcare research.

Future work should also examine how different forms of data use, including cross-sector data linkage and emerging digital data sources, are understood by underrepresented groups, and how context, data type, and governance arrangements shape perceptions of acceptability over time. Researchers should also report participant characteristics and study contexts more consistently to enable future evidence syntheses to examine how social, cultural, and healthcare contexts influence perceptions of big data use. In particular, research should explore governance and consent approaches that support ongoing involvement, transparency, and developmentally appropriate decision-making in paediatric contexts.

### Conclusion

This systematic review provides the first synthesis of evidence on the perceptions, attitudes, and concerns of underrepresented groups regarding the use of big data in CYP healthcare research. Overall, the findings indicate broadly supportive, but conditional, attitudes towards data-driven research. Acceptance appears to be shaped by perceived societal benefit, trust in data users, and the presence of clear safeguards to protect privacy, confidentiality, and data governance. Concerns about data misuse, commercial exploitation, and limited transparency underscore the importance of ethical oversight and meaningful engagement with underrepresented groups. Acceptability depends on whether underrepresented groups recognise the governance, purposes, and power relations around data use as legitimate. Important gaps remain, particularly in relation to the perspectives of ethnic minority populations, younger children under 14 years, and underserved groups in LMIC settings. Addressing these gaps, and embedding inclusive and transparent governance practices, will be essential if big data-driven paediatric research is to be both ethically robust and equitable in the benefits it delivers.

## Supporting information

S1 Checklist PRISMA Checklist. (DOCX)

S1 Text Database search strategy. (PDF)

S2 Text Pre-defined search strategies. (DOCX)

S3 Text Grey literature search strategy. (DOCX)

S1 Table Data extraction table. (PDF)

S2 Table Quality assessment. (DOCX)
