# Supplementary material for: Perceptions, attitudes, and concerns of underrepresented groups regarding the use of big data in children and young people’s healthcare research – A systematic literature review
Source: PLOS Digit Health. 2026 Oct 15;5(10):e0001434. doi: 10.1371/journal.pdig.0001434 (PMC13671531; doi:10.1371/journal.pdig.0001434)
Supplement: Supplementary file 1 [file pdig.0001434.s002.pdf]

MEDLINE (Ovid)

#	Search
**1**	("big data" OR "population data" OR "routinely collected data" OR (health adj2 (data OR record)) OR (medical adj2 record*) OR (patient* adj2 (record* OR data)) OR "personal health information" OR "personal data" OR database* OR dataset* OR "data set*").tw,kf.
**2**	Big Data/ OR Routinely Collected Health Data/ OR Electronic Health Records/ OR Personal Health Records/ OR Medical Records/ OR Records/ OR Medical Records Systems, Computerized OR Databases as Topic/ OR Dataset
**3**	or/1-2
**4**	(“underserved” OR “under-served” OR “underrepresented” OR “under-represented” OR “marginali*” OR “seldom heard” OR “seldom-heard” OR “hard to reach” OR “hard-to-reach" OR “stigmati* population*” OR “minorit*” OR “under 18” OR “parent*” OR “single parent*” OR “guardian*” OR “carer*” OR “religious minorit*” OR “ethnic minorit*” OR “minority ethnic” OR “Black” OR “Asian” OR “BAME” OR “sexual orientation*” OR “LGBT*” OR “gay” OR “lesbian” OR “bisexual*” OR “transgender*” OR “transsexual*” OR “homosexual*” OR “queer” OR “gender-questioning” OR “socio-economically disadvantaged” OR “socioeconomic status” OR “socio-economic status” OR “underprivileged” OR “under-privileged” OR “educational disadvantage*” OR “digital disadvantage” OR “digital exclusion” OR “unemployed” OR “low income” OR “migrant*” OR “transient*” OR “immigrant*” OR “asylum seeker*” OR refugee* OR “travel* communit*” OR “homeless*” OR “no fixed abode” OR “remote area*” OR “language barrier*” OR “prison population*” OR “foster care” OR “foster child*” OR “looked after child*” OR “mental disorder*” OR “mental health” OR “mental* ill*” OR “people with addictions” OR neurodiver* OR “ADHD” OR “Attention Deficit Hyperactivity Disorder” OR “ASD” OR “Autism Spectrum Disorder” OR “lack capacity” OR “cognitive impairment” OR “learning disabilit*” OR “physical disabilit*” OR amputat* or paraplegic* or quadriplegic* OR “visual* impair*” OR blind OR “hearing impair*” OR deaf OR “multiple health conditions” OR “severely ill” OR “chronic health condition*” OR “chronic illness” OR “pregnan*” OR smokers OR obes*).tw,kf.
**5**	Minority Groups/ OR Parents/ OR Single Parent/ OR Caregivers/ OR Black OR Exp Asian People/ OR Ethnic and Racial Minorities/ OR Sexual and Gender Minorities/ OR Transgender Persons/ OR Unemployment/ OR Transients and Migrants/ OR Refugees OR Homeless Youth/ OR Prisoners/ OR Child, Foster/ OR Mental Disorders/ OR Mental Health/ OR Autism Spectrum Disorder OR Attention Deficit Disorder with Hyperactivity OR Exp Learning Disabilities/ OR Exp Disabled Persons/ OR Visually Impaired Persons/ OR Blindness OR Deafness OR Chronic Disease/ OR Pregnant Women/ OR Pregnancy/
**6**	or/4-5
**7**	(share* OR sharing OR transfer* OR access* OR "secondary us*" OR link* OR governance OR mining) adj2 (data OR record* OR information).tw,kf.
**8**	Data Mining/
**9**	or/7-8
**10**	(attitude* OR perspective* OR willing* OR perception* OR barrier* OR enable* OR facilitat* OR opinion* OR trust* OR confiden* OR concern* OR view* OR challenge* OR accept* OR preference*).tw,kf.
**11**	Attitude/ OR Attitude to Health/ OR Health Knowledge, Attitudes, Practice/ OR Trust/ OR Patient preference/
**12**	or/10-11
**13**	(young adj2 ("adult*" OR "individual*" OR "inpatient*" OR "in-patient*" OR "man" OR "men" OR "outpatient*" OR "out-patient*" OR "patient*" OR "people*" OR "person*" OR "population*" OR "wom#n")) OR young OR youngster* OR youth* OR juvenil* OR kid? OR minor? OR Boy* OR girl* OR adolescen* OR teen* OR puber* OR pubescen* OR college* OR underage? OR under-age? OR preteen* OR pre-teen* OR pre-adolescen* OR preadolescen* OR highschool* OR high-school* OR ((high* or secondary) adj2 (education or school*)) OR first-grader* OR second-grader* OR third-grader* OR fourth-grader* OR fifth-grader* OR sixth-grader* OR seventh-grader* OR preschool* OR pre-school* OR kindergarten* OR infan* OR toddler? OR child* OR stepchild* OR step-child* OR babies OR baby OR PICU OR p?ediatric* OR neonat* OR neo-nat* OR newborn* OR new-born* OR perinat*). tw,kf.
**14**	Young Adult/ OR Adolescent/ OR Child, Preschool/ OR Infant/ OR Child/ OR Infant, Newborn/

**15**	or/13-14
**16**	3 AND 6 AND 9 AND 12 AND 15
