# Supplementary material for: Perceptions, attitudes, and concerns of underrepresented groups regarding the use of big data in children and young people’s healthcare research – A systematic literature review
Source: PLOS Digit Health. 2026 Oct 15;5(10):e0001434. doi: 10.1371/journal.pdig.0001434 (PMC13671531; doi:10.1371/journal.pdig.0001434)
Supplement: Supplementary file 2 [file pdig.0001434.s005.pdf]

Underrepresented group	Authors	Overall willingness to share data	Willingness to share what with who	Factors affecting willingness	Perceived benefits / motivators	Concerns / barriers / risks	Conditions for sharing
CYP	Audrey et al., (2016)	Most young people were willing to share their data for research, although young people disagreed whether consent was needed for data linkage.	Not specified	Most (n=34) young people felt consent should be gained for linking teenage pregnancy and state benefits, with one person opposing the research entirely. Most young people (n=36) felt consent should be gained to link birthweight and future health outcomes. An equal number of young people (n=20) felt consent should / shouldn't be gained to link mental health and criminal records. Most young people (n=26) felt consent should be gained to link asthma and postcodes.	Linking birth weight and future health outcomes was seen as for the wider public good. Linking birth weight and future health outcomes was seen as "really important" and for the "greater good". Research into heart disease and asthma were seen as having a clearer purpose and public benefit (compared to research utilising pregnancy and mental health data).	Linking teenage pregnancy with state benefits data: YP perceived there to be a risk of re-identification and being singled out. The potential to offend or stigmatise those on lower incomes Researchers using their sensitive data unknowingly. Linking birth weight and future health outcomes: Whether consent was required for those that has passed. Linking mental health and criminal records: YP felt this data could be potentially stigmatising. The risk of re-identification could threaten their privacy. Potential for biased data due to inaccurate information. Some young men felt this was particularly apparent for this data linkage, where many do not disclose their mental health problems. A lack of trust in data handling procedures. Linking asthma and postcodes: No specific concerns (asthma data was seen as less sensitive).	The data should be anonymised to ensure data privacy. YP were concerned about whether they had full ownership over their data. YP should know if their sensitive data is being accessed and used by researchers. Consent should be sought for healthcare data linkage, to keep people informed about how their data is being used and because it is "fair" / "right".
	Carey et al., (2024)	YP believed that establishing a mental health data bank was acceptable.	Were concerned about sharing their data with private companies. Most trusted researchers and universities as they felt they have good intentions for the data (i.e. to improve mental health and furthering knowledge, respectively). Most trusted charities and nongovernmental organisations (although some mentioned specific organisations that they didn't trust because of a 'track record of stigmatisation or harm'). Wanted to share their data with professionals whose role it is to advocate for the health of young people (i.e. mental health care practitioners).	Not specified	Young people were motivated to share their data by the opportunity to contribute to mental health research. Young people wanted their data to be widely accessible to researchers to allow for more 'research, creativity and discovery.' Empower those with mental health difficulties and improve mental health awareness.	Private companies might misuse their data as they have profit-orientated motives. Distrust in advertising, insurance, and "big pharma" companies to protect their data. Distrust in the media as they may exaggerate or misinterpret findings for profit. Removing barriers (i.e., costs and ID requirements) that could prevent certain groups from accessing the data. The cost of managing and storing the data could prevent researchers with access to less funding from using it. Researchers may have dubious intentions (i.e., eugenics) and their research could have harmful consequences. Concerned about sabotage, corruptions, and destruction of their data further discriminating marginalised groups. Concerned about the use of recreated data and the real-life implications of distorting research findings. Young people expressed concerns about the risk of re-identification. Some were sceptical whether true anonymisation was possible, particularly for small communities. This could be stigmatising. Concerned about how countries with strong societal stigma towards mental health problems or a history of poor human rights may misuse the data. Further, the lack of global laws, language barriers, currency conversion, differences in ID worldwide were concerns.	Young people felt researcher requirements may differ depending on their data needs. Research with potential harm should receive a higher level of scrutiny compared to less harmful projects. Multiple data controllers and data stored in multiple locations. A single data-handler was seen as unacceptably risky. Robust ethical procedures, including ethics training, were perceived as crucial. Privacy, anonymity and confidentiality were seen as essential. Young people felt data managers should be trained in these areas. Young people felt it was important to maintain some control over their data. It should not be accessible to everyone. YP tended to feel that downloading the data was unacceptable. A secure server was seen as more acceptable but may limit researchers conducting their studies in certain parts of the world (i.e., internet connection). Some young people felt that the government has a duty to improve mental health and were obvious funders for a data bank. Universities were generally viewed as trusted institutions with an interest in furthering knowledge as well as having research expertise and thorough ethics review processes.
	Helms et al., (2013)	YP were supportive about the use of linked healthcare data for research, with appropriate data security procedures, access controls, and legal / ethical frameworks.	89.4% of YP felt the NHS could have unrestricted access to children's linked, anonymised healthcare data for research purposes.	YP were more comfortable sharing their data than their parents / guardians.	72% of YP felt data linkage could benefit them as an individual and 74% perceived benefits to wider society. Respondents felt data linkage could benefit them as an individual (72% young people; 91% parents/guardians) and benefit wider society (74% young people; 86% parents/guardians). The majority of the public felt they had a trusting relationship with the NHS, which made them feel more inclined to share their data with this organisation.	YP raised concerns about sharing their data with private companies and about the use of health data for financial rather than direct health gain.	YP expressed a preference for opt-in (12.0%) as opposed to opt-out (10.6%) consent procedures. YP want to be more involved in consent procedures, with the appropriate age of involvement from the age of 13-14 years. 70.4% of YP felt there was a need to obtain consent when using routinely collected data. Most YP (68.2%) felt both YP and parents / guardians should be asked for consent. 44.7% of YP felt health data should be stored for as long as it is useful in advancing health outcomes, with 25.8% of YP perceiving 5 years to be sufficient. 78% of YP thought they had a legal and moral right to decide who can access the data. 50% of YP felt access to health data by non-NHS organisations should be chargable. 89.4% of YP felt the NHS was the most apporpriate guardian of their health data. Anonymisation of data was felt to be important in protecting individual confidentiality.
	Kong et al., (2015)	Most adolescents were willing to donate DNA samples for research purposes, especially if the samples are collected during medical procedures.	Clinic participants (adolescents and parents) were more willing to donate DNA samples compared to school participants in all three scenarios (extra procedure, p.011; extra tube of blood, p<.001; leftover samples, p<.001). 52% of clinic adolescents were willing to donate their DNA data compared to 47% of school adolescents. Adolescents were significantly more likely to say yes to the continued use of samples than parents.	Parents were found to be significantly more likely to say no to DNA donation compared to adolescents. More parents (64% of clinic and 63% of school) felt reconsent was important than adolescents.	Clinic adolescents expressed a keenness "to give back a little." Some school participants expressed a willingness to "support anything that will help children who are sick."	Adolescents expressed concerns about potential discomfort from taking an additional sample, and being fearful of needles / procedures. School adolescents expressed concerns regarding privacy and not knowing exactly what research study their donations would be used for.	Adolescents felt that they are ready for involvement in medical decision-making at age 14.5 years. 18% of clinic and 19% of school adolescents felt patient assent was not necessary. 50% of clinic and 55% of school adolescents felt it was important to gain reconsent. When asked whether samples should still be used if recontact for reconsent was unsuccessful, clinic adolescents were more willing (56%) than school adolescents (49%).
	Sieberts et al., (2023)	Young people were keen to share their anonymised mental health data for research.	Participants from India and South Africa expressed a preference for their data to be used for mental health research only. Participants from the UK reported a slight preference that their data be used for other types of healthcare research.	Enrolment rate did not significantly change by age, gender, or lived experience.	The findings from the databank could help normalise, destigmatise and start conversation around mental health. Could enhance our understanding of mental health and raise awareness.	Having anyone access the data or people from certain places. Hosting the data in on place. Preferred that their data not be used to make a profit and that commercial organisations should pay for access to their data. Data access contracts could be violated (i.e. forged) whereas others perceived these contracts as a useful tool for agreed accountability. Data access fees could prevent smaller research teams with less funds from accessing the data. However, others felt a fee could act as a buy-in and mitigate against potential misuse. If the databank is owned by a private company they may misuse their data for commercial gain. However, others felt it could help financially sustain the databank. Anonymised data still carries a risk of re-identification in small / niche samples. However, participants felt the risk of re-identification was comparable to user tracking (e.g. targeted advertising). Concerns about hackers, bad actors, and historically harmful actors within respected research institutions. Historic untrustworthiness relating to researchers and systems, resulting from previous data misuse / leaks.	In the quantitative study, across sites in India and South Africa, it was seen as most acceptable for a professional review panel to control who has access to the data, an then a democracy, and then volunteer or community review panel. Across the UK site, it was felt a democracy, then professional review panel, ans then volunteer or community panel is most appropriate. In the qualitative study, it was seen as most acceptable for a community review panel to decide who can access the data. In the quantitative study, across sites it was seem as most acceptable to access the data via a secure server, then download, then data steward. In the qualitative study, the most acceptable way to access the data was thought to be via a server, then through a recreated dataset, and then downloaded. In the quantitative study, across sites the researcher norms consent model was seen as least acceptable, with participant choice, democracy/download and democracy/server similiar in acceptability. Ethics training, review board approval, sign contract, provide ID, and pay a fee were considered the ordered steps (from most to least acceptable) prior to gaining data access. The organisation /institution should manage the data, then the government, and then private companies (order of acceptability). Researchers should be restricted to certain types of projects utilising certain types of analyses. Having the "right people" access the data, with the relevant skills / expertise / job / track record. It was viewed as more acceptable to host the data in several locations, not just one.
	Shandhi et al., (2024)	67% of young people were willing to participate in future research studies involving the sharing activity data from smart devices. 29% reported "maybe" and 4% said "no".	Not specified	Willingness to share health data varied significantly across age, with younger generations being more willing to share their health data compared to retirees.	Overall, self-reported measures from health and fitness apps (i.e., mindfulness and mood, water intake, food logs, women's health, etc.) were deemed the least amenable to share for research purposes followed by sleep tracking data.	Not specified	Not specified
	Wild et al., (2023)	Young people had mixed feelings towards data linkage.	Not specified	Not specified	Data sharing could help other children with similiar conditions. Consent discussions could be empowering and enable families to be active participants in their own health journeys.	Individual data points do not represent a whole person. Indigenous ethnic minorities may be overlooked in large datasets and not represented appropriately. Concerns about their data being used may further stigmatise marginalised groups. Their data being inaccessible to them. Lack of trust in institutions and healthcare professionals that the data would be shared with.	Adequate data caretaking (data safety, management and governance). Children felt multiple people should have ownership (i.e. themselves, parents, healthcare professionals). Healthcare professionals should ask to use their data and families should be involved in ongoing consent discussions from childhood and into adulthood. A want to be seen as more than just a number - their data represents a real person behind the numbers. Discriminate sharing (knowing who the data is being shared with and for what purpose) is important.
Ethnic Minority	Halverson & Ross (2012)	Most participants were willing to share their child's genetic data, although a vocal minority opposed.	Most participants were willing to share their child's genetic data with researchers through a biobank.	Not specified	97% reported an interest in receiving individual results relating to their child (less interested in aggregated data).	One parent thought that if they were given access to their child's result, it may be seen as an invasion of their child's privacy. A minority of parents felt their child should be involved in the decision-making process about biobank enrolment, and were hesitant to decide on their behalf. Parents were seen as gatekeepers between their child and researchers.	Most parents felt there isn't / shouldn't be a difference between adult and paediatric biobanking (i.e. ethical considerations are essentially the same). Parents felt they have a right to have control over their child's health data (i.e. use / results etc). Some parents said they'd like to see their child's results, even when they become adults.
Mental Health Service Users	Koposov et al., (2024)	83% felt it is ethical to share their health records for research purposes.	81% were willing to share and trusted health authorities with their health data.	Not specified	93% perceived benefits to data sharing. 84% perceived value in sharing their health information to help others with similiar diagnoses.	Not specified	Not specified

Neurodevelopmental condition	Raspa et al., (2020)	Not specified	Parents of children with FXS or ASD had higher preferences for studies conducted by nonprofit researchers compared to parents of TD children. Parents of children with FXS or ASD had a relatively lower preference for studies conducted by for-profit researchers. Parents of children with ASD had higher preferences for research conducted by government researchers, compared to parents of TD children or those with FXS.	Parent's income, amount of trust in the healthcare system, level of health literacy, and severity of the child's condition as reflected in the number of co-occurring health conditions were associated with the groups who preferred the use of non-identifiable and nonsensitive data (inconsistent across subgroups).	Parents of children with FXS or ASD had higher preferences for studies with a research topic that was personally important to them, compared to parents of TD children. Parents of children with FXS or ASD had higher preferences for studies that would return summary or individual results, compared to parents of TD children.	Parents of children with FXS or ASD had lower preferences for studies that would not return results, compared to parents of TD children, which could present as a barrier to data sharing.	Parents of children with ASD had higher preferences for research using information that is not personally identifiable, compared to parents of TD children and those with FXS. Overall, parents preferred studies that relied on non-identifiable compared to identifiable information. Parents of children with FXS and ASD had lower preferences for studies involving sensitive information compared to parents of TD children; they preferred studies that used non-sensitive over sensitive information. Parents of children with ASD showed higher preferences for studies using nonsensitive information compared to parents of children with FXS, but there was no difference compared to parents of TD children.
Physical health condition	Chow et al., (2024)	Caregivers and youth were generally willing to share their child's healthcare data on the platform.	88.9% of caregivers of a child with a chronic condition agreed with the statement 'I am willing to share my/my child's PHI if it creates progress in nonprofit health research. 72.9% of youth agreed with the same statement.	Not specified	The digital platform was primarily perceived to potentially make it easier to access and share their child's health information. 89.0% of youth and 97.0% of caregivers felt the integrated platform would be helpful to researchers.	91.4% of caregivers and 92.5% of youth felt very or somewhat concerned about web data privacy and security. Primarily concerned that the information they share about their child could be linked to their child in the future and impact job prospects or insurance policies. Also concerned that the information would be sold to third parties and used to make a financial profit.	Not specified
	Hughes et al., (2023)	Were supportive of sharing their routinely collected healthcare data to support CYP's cancer research.	Not specified	Not specified	Perceived value in sharing their data to support research into rare cancers.	Mistrust in data handlers, as a result of negative portrayals of data misuse by the media (i.e. patient data being sold to private companies). Receiving "spam" advertising was seen to lower public confidence - also highlights a lack of awareness of different types of data sharing (i.e., for marketing and for research).	Want to have control over their healthcare data. It is important to inform patients about the secondary use of healthcare data, but were unsure about when is best to inform patients about this (either at diagnosis or some time after). Paediatric patients were happy for their parents to make decisions relating to the use of their healthcare data, but felt as they got older they'd like to be able to make those decisions for themselves. Have transparent data security and anonymisation procedures to raise awareness about the rigorous procedures in place, to build trust.
	Salvaterra et al., (2015)	Most parents agreed to donate the biological material of their children to a biobank (n = 29). Only one mother did not support the enrolment of her child into a biobank.	96.6% of parents were willing to donate their child's biological material to a biobank for research purposes.	Not specified	Some parents perceived their child's involvement in the biobank as 'a good thing to do'. Supporting research development and helping others were the main reasons for their child's involvement in the biobank. Parents whose children had inherited a genetic neurological condition were more motivated to help others, whereas parents whose child acquired a brain injury were more motivated for individual benefits (i.e., quality of life).	Their child's specimens could be used for illegal purposes (i.e., the development of bacteriological arms). Potentially causing physical pain to their child in collecting the specimen. Potential privacy protection breaches. One mother perceived little value in biobank participation, given her son's disorder was caused by a traumatic event. Few perceived individual benefits were a barrier.	Their child's privacy should be protected through the adoption of adequate measures, permitting donor identification when necessary. Their children should be informed about their donation of their specimen to the biobank. Their children should be involved in the initial decision-making processes and/or re-consent. All parents wanted to be informed about the research results.
	Scollon et al., (2014)	80% of parents with children with newly diagnosed cancers had a significant interest in the research and agreed to study enrollment.	94% of parents consented to the use of the diagnostic tumour sample for additional research studies. 95% of parents consented to the collection of tumour for research from any subsequent studies. 94% of parents consented to the collection of an additional 1 to 2 teaspoons of blood for research purposes. 90% of parents consented to the sharing of de-identified genetic and clinical data with other BCM investigators and ARB-approved protocols. 87% of parents consented to the deposition of de-identified genetic and clinical data into a scientific database.	Willingness to participate in clinical genomics research was not significantly impacted by patient-specific factors such as age, gender, race, or ethnicity.	80% of parents with children with newly diagnosed cancers had a significant interest in the research and agreed to study enrollment.	Most commonly, parents (10%) declined to participate in the study due to feeling overwhelmed by their child's new cancer diagnosis, and that they did not wish to participate in additional research studies. 3% of parents expressed concerns about privacy risks. 2.5% reported being anxious about receiving genetic testing results. 2% of parents did not want any further blood being taken for research purposes.	Not specified
	Traore et al., (2015)	Not specified	Not specified	Not specified	Gain access to high quality care with little to no costs. A way to help individual patients receive the appropriate diagnosis and treatment. Parents felt it was an opportunity to check the health status of their child.	There are rumours that blood collected during studies is then sold, demonstrating mistrust in researchers / healthcare professionals. Some parents were comfortable with their data being tested for multiple diseases, but others were concerned about the identification of other diseases (i.e., HIV).	Study information about complex research topics (i.e. geneomics) should be translated into language accessible to participants (such as Bambara). Researchers may benefit from further training to assist with this translation.
	West & Cumella (2018)	Not specified	83.8% of YP were comfortable with sharing their confidential health data for research to develop new asthma treatments. 95.9% of YP were comfortable with sharing their anonymised health data for research to develop a tool to identify people at risk of an asthma attack.	Not specified	Not specified	Not specified	Not specified
Sexual / Gender Minority	Matson et al., (2019)	Most youth were willing to share de-identified blood samples with other researchers.	68.0% were willing to share their blood samples with other researchers.	Black race, feeling that answering questions about sexual behavior and SGM identity was an invasion of privacy, and the belief that the results of the survey could create prejudice against LGBTQ teens was associated with lower willingness to share blood samples. Asian race, sexual experience, and greater comfort with sexual health research procedures was associated with greater willingness to share blood samples.	98.5% reported that it felt good to help other LGBTQ teens through participating in sexual health research. 69.1% felt it would be helpful to share blood samples with other researchers. Youth related a desire to help the community as a reason for their lack of concern regarding data sharing with researchers.	7.1% felt sharing survey results would create prejudice against LGBTQ teens and 2.5% felt it was an invasion of their privacy. The most common concern was the need for confidentiality (including how it would be maintained, protected, and the role of identifiable information) to ensure their privacy was not at risk. Some had fears about their family knowing they've had a HIV test or finding out about their sexual activity or sexual / gender identity. A few were concerned about sharing blood samples given their identifiable nature (i.e., genetic info.) Some expressed a general discomfort with their blood samples being shared (i.e., feels strange). Concerns also related to the future use and long-term intentions with the blood sample data, including whether the longevity of the data could harm them in the future. A few mentioned they would not give blood in the first place due to discomfort with getting blood drawn.	The removal of identifiable information (i.e. names) to protect their confidentiality. Their data should be handled carefully, that researchers should only have access to the data they need, and the disclosure of personal information to the public or their parents is not acceptable. A need to ensure the data is being used as originally intended. Some youth felt consent forms should be administered to obtain permission prior to data sharing.
