# Supplementary material for: Perceptions, attitudes, and concerns of underrepresented groups regarding the use of big data in children and young people’s healthcare research – A systematic literature review
Source: PLOS Digit Health. 2026 Oct 15;5(10):e0001434. doi: 10.1371/journal.pdig.0001434 (PMC13671531; doi:10.1371/journal.pdig.0001434)
Supplement: Supplementary file 4 [file pdig.0001434.s003.docx]

# FILTERS TO RETRIEVE STUDIES RELATED TO UNDERSERVED (MEDICALLY) POPULATIONS

DATABASES: EMBASE, MEDLINE

| EMBASE |  |
| --- | --- |
| **PLATFORM** | OVID |
| **DATABASE** | **EMBASE** |
| **LAST UPDATED DATE** | 09-29-2020 |
| **VALIDATION STATUS** | This filter is not validated |
| **SEARCH FILTER**  (COPY AND PASTE INTO THE EMBASE SEARCH BOX) | transsexualism/ or exp ethnic group/ or bisexuality/ or exp homosexuality or exp disabled person/ or transgender/ or migrant/ or exp refugee/ or exp cultural diversity/ or exp socioeconomics/ or (Metis or Indigenous* or Aboriginal* or Amerindian* or Autochtone* or First Nation* or Inuit or Innu or Inuk or Inuvialuit or tribal or diversity or underprivileged or underrepresented or disadvantaged or minority or minorities or transient* or migrant* or immigrant* or blind or visual* impair* or hearing impair* or deaf or amputat* or paraplegic* or quadraplegic* or wheelchair* or transsexual* or homosexual* or bisexual* or two spirit* or gender-questioning or HIV positive or elders or elderly or oldest old or senior citizen* or shut-in or house-bound or neglected or (battered adj3 (spouse* or wife or wives or partner)) or disable* or transgender* or ethnic* or poverty or impoverished or working poor or unemploy* or under-employed or transportation deficit* or low soci* status or low income* or low-SES or hard-to-house or homeless* or under-housed or (street adj3 (worker* or people or child or children or youth)) or (sex adj3 worker*) or prostitute* or inner city or downtown core or city core or skid row or rural or remote or alcoholic* or mental* ill* or ((drug or substance*) adj3 (abuse* or use* or illegal or illicit or addict* ))).mp. |
| **REFERENCES** | Health Canada. Certain Circumstances Issues in Equity and Responsiveness in Access to Health Care in Canada: A collection of papers and reports prepared for Health Canada, 2001. <https://www.canada.ca/en/health-canada/services/health-care-system/reports-publications/health-care-accessibility/certain-circumstances-issues-equity-responsiveness.html> Accessed Feb 20, 2020.  Simone K, Ahmed RA, Konkin J, Campbell S, Hartling L & Oswald AE. (2018) What are the features of targeted or system-wide initiatives that affect diversity in health professions trainees? A BEME systematic review: BEME Guide No. 50, Medical Teacher, 40:8, 762-780, DOI: 10.1080/0142159X.2018.1473562 |
| **CITATION** | Campbell, SM. Filter to Retrieve Studies Related to Medically Underserved Populations in Canada from the OVID Embase Database. Geoffrey & Robyn Sperber Health Sciences Library, University of Alberta. Rev. September 29, 2020. <https://docs.google.com/document/d/1ePchKXmoaWat1XUAFAyluM1mgdGzorsrvxjZNchYg6Q/edit#heading=h.ldbxqb34y1kj> |

# FILTERS TO RETRIEVE STUDIES RELATED TO UNDERSERVED (MEDICALLY) POPULATIONS

DATABASES: EMBASE, MEDLINE

| MEDLINE |  |
| --- | --- |
| **PLATFORM** | OVID |
| **DATABASE** | **MEDLINE** |
| **LAST UPDATED DATE** | 09-29-2020 |
| **VALIDATION STATUS** | This filter is not validated |
| **SEARCH FILTER**  (COPY AND PASTE INTO THE OVID MEDLINE SEARCH BOX) | Transsexualism/ or exp Ethnic Groups/ or bisexuality/ or exp homosexuality or exp disabled persons/ or transgendered persons/ or "transients and migrants"/ or exp refugees/ or exp Cultural Diversity/ or exp Socioeconomic Factors/ or (Metis or Indigenous* or Aboriginal* or Amerindian* or Autochtone* or First Nation* or Inuit or Innu or Inuk or Inuvialuit or tribal or diversity or underprivileged or underrepresented or disadvantaged or minority or minorities or transient* or migrant* or immigrant* or blind or visual* impair* or hearing impair* or deaf or amputat* or paraplegic* or quadraplegic* or wheelchair* or transsexual* or homosexual* or bisexual* or two spirit* or gender-questioning or HIV positive or elders or elderly or oldest old or senior citizen* or shut-in or house-bound or neglected or (battered adj3 (spouse* or wife or wives or partner)) or disable* or transgender* or ethnic* or poverty or impoverished or working poor or unemploy* or under-employed or transportation deficit* or low soci* status or low income* or low-SES or hard-to-house or homeless* or under-housed or (street adj3 (worker* or people or child or children or youth)) or (sex adj3 worker*) or prostitute* or inner city or downtown core or city core or skid row or rural or remote or alcoholic* or mental* ill* or ((drug or substance*) adj3 (abuse* or use* or illegal or illicit or addict* ))).mp. |
| **REFERENCES** | Health Canada. Certain Circumstances Issues in Equity and Responsiveness in Access to Health Care in Canada: A collection of papers and reports prepared for Health Canada, 2001. <https://www.canada.ca/en/health-canada/services/health-care-system/reports-publications/health-care-accessibility/certain-circumstances-issues-equity-responsiveness.html> Accessed Feb 20, 2020.  Simone K, Ahmed RA, Konkin J, Campbell S, Hartling L & Oswald AE. (2018) What are the features of targeted or system-wide initiatives that affect diversity in health professions trainees? A BEME systematic review: BEME Guide No. 50, Medical Teacher, 40:8, 762-780, DOI: 10.1080/0142159X.2018.1473562 |
| **CITATION** | Campbell, SM. Filter to Retrieve Studies Related to Medically Underserved Populations in Canada from the OVID MEDLINE Database. Geoffrey & Robyn Sperber Health Sciences Library, University of Alberta. Rev. September 29, 2020. <https://docs.google.com/document/d/1ePchKXmoaWat1XUAFAyluM1mgdGzorsrvxjZNchYg6Q/edit#heading=h.qi55eeyvgzy9> |

**Canadian Health Libraries Association**

**Enfant**

Child
Rappel favorisé sur la précision
Stratégie de recherche développée par Vicky Tessier à l'INSPQ puis déposée et mise à jour par Mahée Lacourse.

**Ovid Medline**

Bébés

(babies OR baby OR infan* OR neonat* OR neo-nat* OR newborn* OR new-born* OR perinat*).ti,ab,kf. OR Infant, Extremely Low Birth Weight/ OR Infant, Extremely Premature/ OR Infant, Large for Gestational Age/ OR Infant, Low Birth Weight/ OR Infant, Newborn/ OR Infant, Postmature/ OR Infant, Premature/ OR Infant, Small for Gestational Age/ OR Infant, Very Low Birth Weight/ OR Infant/

Enfants et jeunes

(boy? OR boyfrien* OR boyhood* OR child* OR fifth-grader* OR first-grader* OR fourth-grader* OR girl? OR girlfriend* OR girlhood* OR juvenil* OR kid? OR kindergarten* OR minor? OR minority OR paediatric* OR peadiatric* OR pediatric* OR PICU OR preschool* OR pre-school* OR second-grader* OR seventh-grader* OR sixth-grader* OR stepchild* OR step-child* OR third-grader* OR toddler? OR young OR youngster* OR youth*).ti,ab,kf. OR Child/ OR Child, Preschool/

Adolescents

(adolescen* OR college* OR highschool* OR high-school* OR ((high* OR secondary) ADJ2 (education OR school*)) OR preadolescen* OR pre-adolescen* OR preteen* OR pre-teen* OR puber* OR pubescen* OR teen* OR underage? OR under-age?).ti,ab,kf. OR Adolescent/ OR Minors/

Jeunes adultes

(young ADJ2 (adult* OR individual* OR inpatient* OR in-patient* OR man OR men OR outpatient* OR out-patient* OR patient* OR people* OR person* OR population* OR wom#n)).ti,ab,kf. OR Young Adult/

**EBSCOhost MEDLINE**

Bébés

TI (babies OR baby OR infan* OR neonat* OR neo-nat* OR newborn* OR new-born* OR perinat*) OR AB (babies OR baby OR infan* OR neonat* OR neo-nat* OR newborn* OR new-born* OR perinat*) OR MH ("Infant, Extremely Low Birth Weight" OR "Infant, Extremely Premature" OR "Infant, Large for Gestational Age" OR "Infant, Low Birth Weight" OR "Infant, Newborn" OR "Infant, Postmature" OR "Infant, Premature" OR "Infant, Small for Gestational Age" OR "Infant, Very Low Birth Weight" OR "Infant")

Enfants et jeunes

TI (boy# OR boyfrien* OR boyhood* OR child* OR fifth-grader* OR first-grader* OR fourth-grader* OR girl# OR girlfriend* OR girlhood* OR juvenil* OR kid# OR kindergarten* OR minor# OR minority OR paediatric* OR peadiatric* OR pediatric* OR PICU OR preschool* OR pre-school* OR second-grader* OR seventh-grader* OR sixth-grader* OR stepchild* OR step-child* OR third-grader* OR toddler# OR young OR youngster* OR youth*) OR AB (boy# OR boyfrien* OR boyhood* OR child* OR fifth-grader* OR first-grader* OR fourth-grader* OR girl# OR girlfriend* OR girlhood* OR juvenil* OR kid# OR kindergarten* OR minor# OR minority OR paediatric* OR peadiatric* OR pediatric* OR PICU OR preschool* OR pre-school* OR second-grader* OR seventh-grader* OR sixth-grader* OR stepchild* OR step-child* OR third-grader* OR toddler# OR young OR youngster* OR youth*) OR MH ("Child" OR "Child, Preschool")

Adolescents

TI (adolescen* OR college* OR highschool* OR high-school* OR ((high* OR secondary) N2 (education OR school*)) OR preadolescen* OR pre-adolescen* OR preteen* OR pre-teen* OR puber* OR pubescen* OR teen* OR underage# OR under-age#) OR AB (adolescen* OR college* OR highschool* OR high-school* OR ((high* OR secondary) N2 (education OR school*)) OR preadolescen* OR pre-adolescen* OR preteen* OR pre-teen* OR puber* OR pubescen* OR teen* OR underage# OR under-age#) OR MH ("Adolescent" OR "Minors")

Jeunes adultes

TI (young N2 (adult* OR individual* OR inpatient* OR in-patient* OR man OR men OR outpatient* OR out-patient* OR patient* OR people* OR person* OR population* OR wom?n)) OR AB (young N2 (adult* OR individual* OR inpatient* OR in-patient* OR man OR men OR outpatient* OR out-patient* OR patient* OR people* OR person* OR population* OR wom?n)) OR MH ("Young Adult")

Stratégie développée le 2018-11-30
Ajoutée le 2018-11-30, 15:17 par Mahée Lacourse, révisée par Vicky Tessier le 2023-01-04
