# Supplementary material for: Perceptions, attitudes, and concerns of underrepresented groups regarding the use of big data in children and young people’s healthcare research – A systematic literature review
Source: PLOS Digit Health. 2026 Oct 15;5(10):e0001434. doi: 10.1371/journal.pdig.0001434 (PMC13671531; doi:10.1371/journal.pdig.0001434)
Supplement: Supplementary file 5 [file pdig.0001434.s004.docx]

| Database | Search Strings | Date searched | Number of results | Number of results reviewed |
| --- | --- | --- | --- | --- |
| MedRxiv | (“health data” AND minority AND views AND sharing AND children) | 7/31/2025 | 3,348 | 100 |
|  | (“big data” AND minority AND perceptions AND sharing AND children) | 7/31/2025 | 1,212 | 100 |
|  | (“big data” AND minority AND attitudes AND link AND children) | 7/31/2025 | 774 | 100 |
| PsyArXiv | ("health data" AND share) | 7/31/2025 | 27 | 27 |
|  | ("big data") | 7/31/2025 | 123 | 123 |
|  | (data AND use AND view) | 7/31/2025 | 54 | 54 |
| NIHR Journals Library | ("big data" AND us* AND attitude* AND child* AND minorit*) | 7/31/2025 | 13 | 13 |
|  | ("health data" AND shar* AND view* AND child* AND minorit*) | 7/31/2025 | 10 | 10 |
|  | ("medical record*” AND child* AND perspective* AND minorit*) | 7/31/2025 | 18 | 18 |
| NIHR Open Research | *Searched articles* | 7/31/2025 | 212 | 212 |
|  | *Searched documents* | 7/31/2025 | 89 | 89 |
| The Kings Fund Library Database | ("big data" AND attitude) | 7/31/2025 | 1 | 1 |
|  | ("health data" AND shar*) | 7/31/2025 | 58 | 58 |
|  | ("health data" AND minorit*) | 7/31/2025 | 12 | 12 |
|  | ("medical record*" AND minorit*) | 7/31/2025 | 2 | 2 |
| The Kings Fund Digital Archive | (“big data” AND share AND view AND child*) | 7/31/2025 | 65 | 65 |
|  | (“health data” AND share AND perspective* AND child*) | 7/31/2025 | 79 | 79 |
|  | (“health data” AND us* AND attitude* AND child*) | 7/31/2025 | 41 | 41 |
| Library Hub Discover | ("health data" AND shar*) | 7/31/2025 | 16 | 16 |
|  | ("health data" AND us*) | 7/31/2025 | 36 | 36 |
|  | ("medical records" AND shar*) | 7/31/2025 | 9 | 9 |
| Google Scholar | ("big data" AND minorit* AND view* AND shar* AND child*) | 7/31/2025 | 17,700 | 100 |
|  | (health data AND minority* AND perspective* AND share* AND child*) | 7/31/2025 | 27,700 | 100 |
|  | ("health data" AND attitude* AND minority* AND shar* AND child*) | 7/31/2025 | 15,900 | 100 |
|  | ("big data" AND view* AND minority* AND us* AND child*) | 7/31/2025 | 38,100 | 100 |
| [BASE](https://www.base-search.net/) | ("big data" AND minorit* AND view* AND shar* AND child*) | 4/9/2025 | 10 | 10 |
|  | (health data AND minority* AND perspective* AND share* AND child*) | 4/9/2025 | 153 | 100 |
|  | (health data AND minorit* AND view* AND share* AND child*) | 4/9/2025 | 179 | 100 |
| [OATD](https://oatd.org/) | (“big data” AND share* AND view* AND child*) | 4/9/2025 | 3 |  |
|  | (“health data” AND shar* AND perspective* AND child*) | 4/9/2025 | 3 |  |
|  | (“health data” AND us* AND attitude* AND child*) | 4/9/2025 | 4 | 4 |
|  | (“health data” AND (view*) AND ("child*") AND (minorit*) | 4/9/2025 | 43 | 43 |
| House of Commons Library - Health | health data AND "minorit*" AND "perspective*" AND "share*" AND "child*" |  | 0 | 0 |
|  | “health data” AND shar* AND perspective* AND child* | 4/9/2025 | 0 | 0 |
|  | “health data” AND shar* | 4/9/2025 | 0 | 0 |
|  | “health data” AND child* | 4/9/2025 | 1 | 1 |
|  | ("big data" AND view* AND minorit* AND us* AND child*) | 4/9/2025 | 0 | 0 |
|  | ("big data" AND view* AND minorit* AND shar* AND child*) | 4/9/2025 | 0 | 0 |
|  | “big data” AND child* | 4/9/2025 | 3 | 3 |
| [NESTA](https://www.nesta.org.uk/) | ("big data" AND view* AND minorit* AND shar* AND child*) |  | 7416 | 100 |
|  | ("big data" AND perspectiv* AND minorit* AND shar* AND child*) | 4/9/2025 | 7415 | 100 |
|  | "health data" AND "minorit*" AND "perspective*" AND "share*" AND "child*" | 4/9/2025 | 7424 | 100 |
|  | ("health data" AND minorit* AND view* AND share* AND child*) | 4/9/2025 | 7424 |  |
| [NHS England - Transformation Directorate](https://transform.england.nhs.uk/) |  | 4/9/2025 | N/A | N/A |
| [Patient Experience Library](https://www.patientlibrary.net/cgi-bin/library.cgi) | "health data" AND minority | 4/9/2025 | 1,196 | 100 |
|  | "health record*" AND child* | 4/9/2025 | 1,256 | 100 |
|  | "big data" AND shar* | 4/9/2025 | 262 | 100 |
| [Wellcome Open Research](https://wellcomeopenresearch.org/) | "health data" AND "share" AND "perspective" | 5/9/2025 | 1 | 1 |
|  | "health data" AND "share" AND "child" | 5/9/2025 | 1 | 1 |
|  | "health records" AND "share" | 5/9/2025 | 1 | 1 |
