# Supplementary material for: Perceptions, attitudes, and concerns of underrepresented groups regarding the use of big data in children and young people’s healthcare research – A systematic literature review
Source: PLOS Digit Health. 2026 Oct 15;5(10):e0001434. doi: 10.1371/journal.pdig.0001434 (PMC13671531; doi:10.1371/journal.pdig.0001434)
Supplement: Supplementary file 6 [file pdig.0001434.s006.docx]

| **MMAT Criteria** | **Articles reporting quantitative data** | Kong et al., (2015) | Koposov et al., (2024) | Raspa et al., (2020) | Scollon et al., (2014) | Shandhi et al., (2024) | **Articles reporting qualitative data** | Audrey et al., (2016) | Carey et al., (2024) | Hughes et al., (2023) | Salvaterra et al., (2015) | Traore et al., (2015) | Wild et al., (2023) | **Articles reporting quantitative and qualitative data** | Chow et al., (2024) | Halverson & Ross (2012) | Matson et al., (2019) | Sieberts et al., (2023) |
| --- | --- | --- | --- | --- | --- | --- | --- | --- | --- | --- | --- | --- | --- | --- | --- | --- | --- | --- |
| Clear research questions |  |  |  |  |  |  |  |  |  |  |  |  |  |  |  |  |  |  |
| Data addresses the research question |  |  |  |  |  |  |  |  |  |  |  |  |  |  |  |  |  |  |
| **Qualitative studies** | | | | | | | | | | | | | | | | | | |
| 1.1 Qualitative approach appropriate |  |  |  |  |  |  |  |  |  |  |  |  |  |  |  |  |  |  |
| 1.2 Adequate data collection methods |  |  |  |  |  |  |  |  |  |  |  |  |  |  |  |  |  |  |
| 1.3 Findings adequately derived from the data |  |  |  |  |  |  |  |  |  |  |  |  |  |  |  |  |  |  |
| 1.4 Results sufficiently substantiated by the data |  |  |  |  |  |  |  |  |  |  |  |  |  |  |  |  |  |  |
| 1.5 Coherence between qualitative data sources, collection, analysis and interpretation |  |  |  |  |  |  |  |  |  |  |  |  |  |  |  |  |  |  |
| **Quantitative randomized controlled trials** | | | | | | | | | | | | | | | | | | |
| 2.1 Randomization appropriately performed |  |  |  |  |  |  |  |  |  |  |  |  |  |  |  |  |  |  |
| 2.2 Comparable groups at baseline |  |  |  |  |  |  |  |  |  |  |  |  |  |  |  |  |  |  |
| 2.3 Complete outcome data |  |  |  |  |  |  |  |  |  |  |  |  |  |  |  |  |  |  |
| 2.4 Outcome assessors blinded to the intervention provided |  |  |  |  |  |  |  |  |  |  |  |  |  |  |  |  |  |  |
| 2.5 Participants adhered to the assigned intervention |  |  |  |  |  |  |  |  |  |  |  |  |  |  |  |  |  |  |
| **Quantitative non-randomized** | | | | | | | | | | | | | | | | | | |
| 3.1 Participants representative of the target population |  |  |  |  |  |  |  |  |  |  |  |  |  |  |  |  |  |  |
| 3.2 Appropriate measurements regarding both the outcome and intervention |  |  |  |  |  |  |  |  |  |  |  |  |  |  |  |  |  |  |
| 3.3 Complete outcome data |  |  |  |  |  |  |  |  |  |  |  |  |  |  |  |  |  |  |
| 3.4 Confounders accounted for in the design and analysis |  |  |  |  |  |  |  |  |  |  |  |  |  |  |  |  |  |  |
| 3.5 Intervention administered as intended |  |  |  |  |  |  |  |  |  |  |  |  |  |  |  |  |  |  |
| **Quantitative descriptive** | | | | | | | | | | | | | | | | | | |
| 4.1 Sampling strategy relevant to address the research question |  |  |  |  |  |  |  |  |  |  |  |  |  |  |  |  |  |  |
| 4.2 Sample representative of the target population |  |  |  |  |  |  |  |  |  |  |  |  |  |  |  |  |  |  |
| 4.3 Appropriate measurements |  |  |  |  |  |  |  |  |  |  |  |  |  |  |  |  |  |  |
| 4.4 Low risk of nonresponse bias |  |  |  |  |  |  |  |  |  |  |  |  |  |  |  |  |  |  |
| 4.5 Appropriate statistical analysis to answer the research question |  |  |  |  |  |  |  |  |  |  |  |  |  |  |  |  |  |  |
| **Mixed methods** | | | | | | | | | | | | | | | | | | |
| 5.1 Adequate rationale for using a mixed methods design |  |  |  |  |  |  |  |  |  |  |  |  |  |  |  |  |  |  |
| 5.2 Effectively integrated different components of the study |  |  |  |  |  |  |  |  |  |  |  |  |  |  |  |  |  |  |
| 5.3 Adequate interpretation of quantitative and qualitative components |  |  |  |  |  |  |  |  |  |  |  |  |  |  |  |  |  |  |
| 5.4 Adequately addressed divergences and inconsistencies between quantitative and qualitative results |  |  |  |  |  |  |  |  |  |  |  |  |  |  |  |  |  |  |
| 5.5 Different components of the study adhere to the quality criteria |  |  |  |  |  |  |  |  |  |  |  |  |  |  |  |  |  |  |

| **Key** | |
| --- | --- |
|  | Yes |
|  | Can’t tell |
|  | No |
