# Supplementary material for: Perceptions, attitudes, and concerns of underrepresented groups regarding the use of big data in children and young people’s healthcare research – A systematic literature review
Source: PLOS Digit Health. 2026 Oct 15;5(10):e0001434. doi: 10.1371/journal.pdig.0001434 (PMC13671531; doi:10.1371/journal.pdig.0001434)
Supplement: Supplementary file 7 [file pdig.0001434.s008.docx]

Dear Dr Bernasconi, Dr Kuo, and Dr Celi,

Thank you for your letter dated 20th July 2026 regarding the manuscript PDIG-D-26-00740, titled *“Perceptions, attitudes, and concerns of underrepresented groups regarding the use of big data in children and young people’s healthcare research – a systematic literature review”.*

We are grateful to you and to the reviewers for the careful reading of our manuscript and for the thoughtful and constructive comments provided. We have carefully considered all of the points raised and have revised the manuscript accordingly.

In particular, we have clarified aspects of the review methodology, including the search strategy, study selection, quality appraisal, and data extraction processes. We have also expanded the *Discussion* and *Strengths and Limitations* sections to provide greater consideration of the heterogeneity and context of the evidence base, including the uneven representation of underserved groups, the use of parental or caregiver perspectives as proxies for CYP, potential selection bias, and methodological limitations within the included studies. We have made further amendments throughout the manuscript to improve clarity and reduce repetition.

For ease of review, we have highlighted changes within the revised manuscript using tracked changes. Below, we provide a point-by-point response to each reviewer comment, indicating where the corresponding changes have been made in the revised manuscript.

We thank you and the reviewers again for the time and care given to reviewing our manuscript. We believe that the revisions have helped to clarify the scope and interpretation of the review findings.

Yours sincerely,

Charlotte Burdge
on behalf of all the co-authors

We thank the reviewers for their comments. Please find our responses listed below:

**# Reviewer 1**

**R1.1**

**Definition and heterogeneity of “big data”. The definition of “big data” remains very broad and encompasses highly heterogeneous contexts (EHRs, biobanks, genetic data, smart devices, data linkage, mental health data). This heterogeneity risks reducing the conceptual coherence of the review. A more explicit discussion of the ethical and perceptual differences between these data contexts would strengthen the interpretation of the findings, as participants may react very differently depending on the specific type of data and research context considered. In addition, lines 285–287 state: “Big data in healthcare research was defined as large-scale healthcare datasets used for research purposes, including electronic health records, administrative health data, and linked healthcare datasets.” A reference supporting this definition appears to be missing.**

Thank you for this helpful comment. We have added a supporting reference for our definition of big data in healthcare research.

*“This definition is consistent with descriptions of big data in healthcare as large, complex, electronic health datasets (46).”*

We have also expanded the *Phenomenon of Interest* section to provide examples of how different healthcare data contexts may raise distinct ethical and perceptual considerations.

*“For example, secondary use of electronic health records, prospective donation of biological samples, linkage of administrative datasets, and use of mental health or smart device data for research may raise different questions relating to privacy, consent, identifiability, governance, and the perceived sensitivity of the data.”*

Finally, we have revised the *Strengths and Limitations* section to acknowledge that variation in data types and research contexts may influence participants' perceptions and limit comparability across studies. Given the limited evidence base, we did not consider it meaningful to synthesise perceptions according to specific data types, and have therefore clarified that the findings should be interpreted cautiously.

*“Furthermore, given the limited evidence base and the heterogeneity of the included data contexts, it was not considered meaningful to synthesise perceptions according to specific data types. Consequently, findings should be interpreted cautiously, recognising that attitudes may vary according to the type of health data and research context considered.”*

**R1.2**

**Limited geographical representation and heterogeneity of underserved groups. Only 17 studies were included out of more than 10,000 screened records, resulting in a relatively limited evidence base. The geographical distribution is heavily skewed toward high-income Anglophone countries (7 UK studies, 4 US, 2 Canadian), while LMIC settings are almost entirely absent. Consequently, conclusions regarding “conditional support” are largely derived from high-income healthcare contexts.**

**Furthermore, the category of “underrepresented groups” includes highly heterogeneous populations — healthy adolescents, oncology patients, youth with FXS/ASD, ethnic minorities, sexual and gender minorities, and mental health service users — with very different social experiences, relationships with healthcare institutions, and historical experiences of research participation. The aggregated synthesis may therefore flatten important distinctions between groups. In particular:
• only one study explored ethnic minority perspectives;
• only one study focused on sexual and gender minority youth;
• only one study examined mental health service users.**

**The claim of systematically examining the perspectives of “underrepresented groups” is therefore only partially supported by the currently available evidence. Recommendation: I would encourage the authors to include a more explicit discussion of the uneven evidence distribution across groups and to clarify how many conclusions in the synthesis rely on only one or two studies.**

Thank you for taking the time to highlight this important limitation of our study, and for suggesting ways in which we could acknowledge it more explicitly. We agree that the current evidence base is heavily weighted towards high-income Anglophone countries and provides limited representation of several underserved groups. While this is indicative of the state of the literature available during the conduct of this review, we also agree that these limitations should be made more explicit within the manuscript. The number of studies contributing to individual findings also varied, with some findings reported in only one or two studies, reflecting the uneven distribution of evidence across the included populations. We have therefore made this limitation explicit, but have not quantified the contribution of individual studies to each finding, as this was a narrative synthesis and the number of studies reporting a finding was not used to determine its salience or weight within the synthesis. Accordingly, we have added the following text to the *Strengths and Limitations* section:

*“Another significant barrier to generalisability stems from the limited number of studies conducted with underrepresented groups, meaning that these perspectives may be further marginalised in our review. For example, only one study each included ethnic minority perspectives, sexual and gender minority CYP, and mental health service users. The synthesis therefore potentially risks an overrepresentation of perspectives from CYP who might not experience the same barriers to healthcare research participation as their more marginalised peers. Any conclusions drawn from the synthesis should be interpreted with a contextual understanding of the structural differences that underrepresented groups experience when it comes to engaging with healthcare institutions and research conducted in such contexts.”*

**R1.3**

**Reliance on parental proxy perspectives. In 6 of the 17 included studies, perspectives were exclusively parental rather than directly reported by CYP. One additional study included mixed perspectives. Therefore, the abstract’s claim of examining CYP perspectives is somewhat overstated. Recommendation: Please discuss more explicitly the methodological implications of relying on proxy perspectives. It may also be worth revisiting the wording of the abstract and potentially the title to better reflect the predominance of parental voices in part of the evidence base.**

Thank you for this comment. We have revised the abstract to more clearly report the number of CYP and parents/carers represented across the included studies and clarified that one mixed study did not report participant numbers separately by group.

*“Seventeen studies were included, capturing perspectives from 6261 participants, including at least 4710 CYP and 1443 parents and carers; one study did not report participant numbers separately by group.”*

We have also expanded the Discussion section to acknowledge the methodological implications of relying on parental or caregiver proxy perspectives, noting that these may not fully reflect CYP's own views and that the findings should therefore be interpreted as representing the perspectives of both CYP and proxy decision-makers rather than CYP alone.

*“Third, the perspectives of younger children, particularly those under 14 years, were rarely represented. Six of the seventeen included studies focused on parental or caregiver perspectives acting as proxy decision-makers, which may not fully reflect CYP's own views. Although this may reflect practical and ethical challenges in conducting research with younger participants (16), it limits understanding of how younger children conceptualise privacy, trust, and participation in research involving their health data. Consequently, the findings should be interpreted as representing the perspectives of both CYP and proxy decision-makers rather than CYP alone.”*

We also considered revising the title but felt it remained appropriate, as the review was designed to examine the perspectives of underrepresented groups regarding the use of big data in CYP healthcare research, rather than CYP perspectives alone.

**R1.4**

**Selection bias and participant recruitment. Most included studies recruited participants in clinical settings or among individuals already engaged with healthcare or research systems. Participants willing to engage in focus groups or research discussions about health data are likely to have more favourable attitudes toward research participation than underserved populations more broadly. The observed “conditional support” may therefore partly reflect selection bias rather than a robust estimate of real-world attitudes. This issue is currently underdeveloped in the manuscript. Recommendation: Please add a more explicit discussion of potential selection bias in the Strengths and Limitations section.**

Thank you for raising this important limitation relating to the recruitment strategies of the studies included in this review. We agree that this issue warrants more explicit discussion, particularly given the potential influence of selection bias on findings relating to willingness and conditional support. Accordingly, we have added the following text to the existing paragraph in the *Strengths and Limitations* section discussing the gap between expressed willingness and real-world participation in research:

*“This is particularly salient considering most of the studies included in this review recruited populations who were already engaged in clinical or healthcare research processes, thereby potentially reflecting a preexisting willingness or favourability towards research participation. Therefore, it is important to contextualise findings on conditional support and willingness by considering the likely effect of selection bias stemming from such recruitment strategies.”*

**R1.5**

**Several sections of the Discussion become somewhat repetitive, particularly regarding the concept of “conditional support”. Greater synthesis and conciseness would improve readability.**

Thank you for this helpful suggestion. We have revised the *Discussion* section to improve conciseness and reduce repetition, particularly in relation to the concept of conditional support.

**R1.6**

**Minor grammatical and stylistic issues:
• “data are routinely reused beyond its original purpose” → “their original purpose”
• “governance structures that is visible” → “governance structures that are visible”**

Thank you for highlighting these grammatical and stylistic issues. We have corrected both in the revised manuscript.

**R1.7**

**Please ensure that abbreviations are defined at first use unless universally recognised. Some abbreviations appear before being defined (e.g., UNCRC, NIHR, FXS, ASD).**

Thank you for bringing this to our attention. We have reviewed the manuscript to ensure that abbreviations, including UNCRC, NIHR, FXS, and ASD, are defined at first use where appropriate.

**R1.8**

**The statement: “Perspectives could be reported directly by CYP, defined as individuals aged 0–25 years” raises a conceptual question. Why were participants older than the age of legal majority included within the CYP category? A brief justification would be useful.**

Thank you for this comment. We have clarified that the definition of CYP (0-25 years) was adopted in alignment with NHS guidance, which commonly defines CYP as individuals up to 25 years of age.

*“Perspectives could be reported directly by CYP, defined as individuals aged 0-25 years in line with the UK’s National Health Service (NHS) guidance recognising young people up to 25 years of age (45), or by parents, carers, or guardians acting as proxy decision-makers in relation to the use of CYP healthcare data.”*

**R1.9**

**Lines 273–276 repeat content already presented in lines 169–173 regarding the NIHR-INCLUDE framework definition.**

Thank you for this suggestion. We carefully considered this recommendation but have retained the definition in both places, as the two instances serve different purposes. The first introduces the NIHR INCLUDE framework in the Introduction, whereas the second defines the eligibility criteria in the Methods. We felt this improved clarity for readers.

**R1.10**

**Line 336: “Discrepancies were resolved through discussion.” Was a third reviewer involved when consensus could not be reached?**

Thank you for raising this important point. We have clarified the grey literature screening procedure by explicitly stating that discrepancies were resolved through discussion between the two reviewers until consensus was reached, and that a third reviewer was not required.

*“Discrepancies were resolved through discussion between the two reviewers until consensus was reached; a third reviewer was therefore not required.”*

**R1.11**

**Lines 350–352: “Two authors (CB/LZ) appraised a subset of articles (n = 5) jointly…”. The description of how quality appraisal was subsequently divided between reviewers is somewhat unclear and may benefit from clarification.**

Thank you for flagging this important point. We have clarified the quality appraisal procedure to explain that the first five articles were independently appraised by both CB and LZ to ensure consistency, after which the remaining articles were appraised by either reviewer, with any queries resolved through discussion until consensus was reached.

*“Two authors (CB/LZ) independently appraised a subset of articles (n = 5) to ensure consistency and reduce the risk of bias. The remaining articles (n = 12) were then assessed by either CB or LZ, with regular discussions to resolve queries.”*

**R1.12**

**There are several inconsistencies in decimal notation throughout the manuscript (commas and decimal points).**

Thank you for highlighting this. We have reviewed the manuscript and standardised decimal notation throughout, ensuring consistent use of decimal points.

**R1.13**

**Line 440: “people believed that the NHS should have unrestricted access…”
Please clarify “NHS” for international readers.**

Thank you for highlighting this. We have revised the text to define the UK National Health Service (NHS) at first mention for the benefit of international readers.

**R1.14**

**Line 987: “Only studies reported in English were included…”. Please clarify whether this was due to language limitations, feasibility constraints, or lack of non-English literature identified.**

Thank you for bringing this to our attention. We have now clarified in line 253 that no geographical restrictions were applied, but studies had to be reported in English due to language limitations within the authorship team. We have also specified the impact this may have on generalisability of this study’s findings. In lines 1022 to 1026 we have outlined the following limitations from this decision:

*“Only studies reported in English were included due to language limitations within the authorship team, which may limit generalisability. This restriction may also have contributed to the predominance of studies conducted in Anglophone high-income countries (14 of 17 studies), particularly the UK, with limited representation of studies from low- and middle-income countries (LMICs) and the Global South.”*

**R1.15**

**Table 1: “Eligibility Citeria” should read “Eligibility Criteria”.**

Thank you for flagging this typo; we have now corrected the spelling to ‘criteria’ on line 257 of the manuscript.

**R1.16**

**No studies were excluded based on quality appraisal scores. Please discuss more explicitly how lower-quality studies may have influenced the certainty and robustness of the conclusions.**

Thank you for this comment. We have expanded the *Strengths and Limitations* section to clarify the implications of retaining all eligible studies in line with MMAT guidance. Specifically, we now acknowledge that several included studies either did not meet one or more appraisal criteria or provided insufficient information for one or more criteria to be assessed, and discuss how these methodological limitations and reporting uncertainties may reduce confidence in some findings contributing to the synthesis.

*“Third, in line with MMAT guidance (49), no studies were excluded on the basis of methodological quality, as the MMAT discourages calculating overall appraisal scores. Nevertheless, nine included studies either did not meet one or more appraisal criteria or provided insufficient information for one or more criteria to be assessed. Consequently, findings from these studies should be interpreted with appropriate caution, as methodological limitations and insufficient reporting may reduce confidence in the reliability of some findings contributing to the synthesis.”*

**# Reviewer 2**

**R2.1**

**I believe that you should more clearly highlight why you focused this research on the combined population of children and young people, but not divide them into two different groups.**

Thank you for this helpful suggestion. We considered stratifying the findings by CYP separately. However, the review was designed to examine perceptions relating to big data in CYP healthcare research, reflecting the terminology commonly used within healthcare policy and research. In addition, the limited and heterogeneous evidence base did not support meaningful synthesis according to age group, as relatively few studies focused exclusively on younger children. We have therefore retained the combined CYP population while acknowledging in the *Discussion* section that the perspectives of younger children, particularly those under 14 years, remain underrepresented and warrant further research.

*“Third, the perspectives of younger children, particularly those under 14 years, were rarely represented, limiting the extent to which findings could be meaningfully examined separately by age group.”*

**# Reviewer 3**

**R3.1**

**One area that this paper could be strengthened is to organize and emphasize the cross-sectional differences in the findings. This is already partially done, as noted above, but I believe reorganizing the discussion to directly address these differences would be valuable. For example, the section "Concerns and Privacy Risks" and paragraph around line 741 read as a laundry list of issues without fully examining who has which issues. By grouping everyone together, this organization undermines the authors' argument that these populations are heterogeneous and can have different concerns. I understand the limitations of the search, which did not cover a lot of the Global South, but I feel discussing these limitations alongside illustrative examples of intersectional attitudes in different contexts will do a good job of motivating the authors' call for more direct research exploring the attitudes of underrepresented populations (line 911).**

Thank you for taking the time to review our manuscript, and for your recommendation to highlight differences between groups and contexts in the findings. Your recommendation is particularly salient to us considering the restriction to studies reported in English which resulted in most included studies coming from Anglophone countries. We also echo your recommendation for a more intersectional approach to the differences that emerged during the synthesis process; however, we feel that reorganising the paper to highlight these differences is beyond the scope of this review, given the paucity of evidence from the Global South, and several specific underrepresented groups, including ethnic minority groups (one study), sexual and gender minority CYP (one study), and mental health service users (one study). Nevertheless, in light of your recommendation and that of Reviewer 1, we have expanded the *Strengths and Limitations* section to highlight the risk of overrepresentation in this review of perspectives from CYP who live with fewer intersecting marginalised identities, as described in our response to R1.2.

**R3.2**

**I also feel that the authors should spend more time addressing the context of the included papers. While the authors point out that there are different types of underrepresented groups, each with their own potential concerns around data usage, the position of underrepresentation is context-specific. An ethnic group which is underrepresented in one context is not so in another. I discussing the findings, I would appreciate more detail about not only the nature of the group expressing the concern, but also the setting that they are in.**

Thank you for this thoughtful comment. We agree that underrepresentation is context-dependent and that the experiences and concerns of different groups are shaped by the settings in which research takes place. Study setting, sampling approach, participant characteristics, and healthcare data type were therefore extracted and are reported in the study characteristics table (*S5 File*). However, given the heterogeneity of the included populations, countries, healthcare systems, and research contexts, we did not consider that a more detailed contextual synthesis could be undertaken without over-interpreting the available evidence. We have therefore retained the contextual information reported for individual studies while acknowledging this limitation and have expanded the *Recommendations for Future Research* section to encourage more consistent reporting of participant characteristics and study contexts.

*“Researchers should also report participant characteristics and study contexts more consistently to enable future evidence syntheses to examine how social, cultural, and healthcare contexts influence perceptions of big data use.”*

**R3.3**

**Along those lines, I would appreciate more reflection on the methodology of this search. In particular, it's not clear to me how the term lists for the search were constructed, and as this term list is designed to catch research about "underrepresented groups," I'm left wondering how well it does so and whether it does so from the perspective of the context of the authors. I would also appreciate some detail on who is involved in the screening and coding process and what efforts the authors made to improve the reliability of their process.**

Thank you for this comment. We have clarified the development of the search strategy and the processes used to ensure consistency and reliability throughout the review. The search strategy was developed using multiple approaches, including search terms informed by the NIHR-INCLUDE framework, supplemented with additional keywords to capture underserved populations not explicitly represented within the framework, and adaptation of established search strategies developed by the Canadian Health Libraries Association and the University of Alberta. We have also added detail to the *Sample* section of the *Methods* to acknowledge the context-dependent nature of whether a population is considered underserved:

*“We recognise that whether a population is considered underserved is context-dependent; eligibility was therefore determined through application of the predefined inclusion criteria rather than by search terms alone.”*

We have also clarified the screening, quality appraisal, and data extraction procedures in the *Methods* section, including the processes used to ensure consistency and reach consensus.

In the *Study Selection and Data Extraction* section, we have clarified that discrepancies in grey literature screening were resolved between the two reviewers:

*“Discrepancies were resolved through discussion between the two reviewers until consensus was reached; a third reviewer was therefore not required.”*

In the *Quality Appraisal* section, we have clarified how articles were allocated between reviewers:

*“Two authors (CB/LZ) independently appraised a subset of articles (n = 5) to ensure consistency and reduce the risk of bias. The remaining articles (n = 12) were then assessed by either CB or LZ, with regular discussions to resolve queries.”*
