# Supplementary material for: Perceptions, attitudes, and concerns of underrepresented groups regarding the use of big data in children and young people’s healthcare research – A systematic literature review
Source: PLOS Digit Health. 2026 Oct 15;5(10):e0001434. doi: 10.1371/journal.pdig.0001434 (PMC13671531; doi:10.1371/journal.pdig.0001434)
Supplement: Peer review history

# Peer review: Decision Letter 0

**DOI:** 10.1371/journal.pdig.0001434.r001

**Authors:** Andrea Bernasconi, Po-Chih Kuo

20 Jul 2026

Response to Reviewers Revised Manuscript with Track Changes Manuscript **Journal Requirements:**

Please confirm at this time whether or not your submission contains all raw data required to replicate the results of your study. Authors must share the “minimal data set” for their submission. PLOS defines the minimal data set to consist of the data required to replicate all study findings reported in the article, as well as related metadata and methods (https://journals.plos.org/plosone/s/data-availability#loc-minimal-data-set-definition).

For example, authors should submit the following data:

- The values behind the means, standard deviations and other measures reported;

- The values used to build graphs;

- The points extracted from images for analysis.

Authors do not need to submit their entire data set if only a portion of the data was used in the reported study.

If your submission does not contain these data, please either upload them as Supporting Information files or deposit them to a stable, public repository and provide us with the relevant URLs, DOIs, or accession numbers. For a list of recommended repositories, please see https://journals.plos.org/plosone/s/recommended-repositories.

**Additional Editor Comments (if provided):** **Reviewers' Comments:**

**Comments to the Author**

1. Does this manuscript meet PLOS Digital Health’s publication criteria?

Reviewer #1: Yes

Reviewer #2: Yes

Reviewer #3: Yes

**********

2. Has the statistical analysis been performed appropriately and rigorously?

Reviewer #1: Yes

Reviewer #2: Yes

Reviewer #3: N/A

**********

3. Have the authors made all data underlying the findings in their manuscript fully available (please refer to the Data Availability Statement at the start of the manuscript PDF file)?

The PLOS Data policy

Reviewer #1: Yes

Reviewer #2: Yes

Reviewer #3: Yes

**********

4. Is the manuscript presented in an intelligible fashion and written in standard English?

Reviewer #1: Yes

Reviewer #2: Yes

Reviewer #3: Yes

**********

**Reviewer #1** : Dear Authors,

Thank you for the opportunity to review this manuscript. I believe this review addresses an important and timely gap in the literature regarding the perceptions of underrepresented groups toward the use of big data in paediatric healthcare research.

The manuscript is well written, methodologically rigorous, and provides a valuable contribution to the literature. The narrative synthesis is rich and nuanced, and the focus on ethics, trust, governance, and acceptability is particularly important in the context of health research.

However, I have several comments that I believe could further strengthen the manuscript.

Major comments

1. Definition and heterogeneity of “big data”

The definition of “big data” remains very broad and encompasses highly heterogeneous contexts (EHRs, biobanks, genetic data, smart devices, data linkage, mental health data). This heterogeneity risks reducing the conceptual coherence of the review. A more explicit discussion of the ethical and perceptual differences between these data contexts would strengthen the interpretation of the findings, as participants may react very differently depending on the specific type of data and research context considered.

In addition, lines 285–287 state:

“Big data in healthcare research was defined as large-scale healthcare datasets used for research purposes, including electronic health records, administrative health data, and linked healthcare datasets.”

A reference supporting this definition appears to be missing.

2. Limited geographical representation and heterogeneity of underserved groups

Only 17 studies were included out of more than 10,000 screened records, resulting in a relatively limited evidence base. The geographical distribution is heavily skewed toward high-income Anglophone countries (7 UK studies, 4 US, 2 Canadian), while LMIC settings are almost entirely absent. Consequently, conclusions regarding “conditional support” are largely derived from high-income healthcare contexts.

Furthermore, the category of “underrepresented groups” includes highly heterogeneous populations — healthy adolescents, oncology patients, youth with FXS/ASD, ethnic minorities, sexual and gender minorities, and mental health service users — with very different social experiences, relationships with healthcare institutions, and historical experiences of research participation. The aggregated synthesis may therefore flatten important distinctions between groups.

In particular:

• only one study explored ethnic minority perspectives;

• only one study focused on sexual and gender minority youth;

• only one study examined mental health service users.

The claim of systematically examining the perspectives of “underrepresented groups” is therefore only partially supported by the currently available evidence.

Recommendation: I would encourage the authors to include a more explicit discussion of the uneven evidence distribution across groups and to clarify how many conclusions in the synthesis rely on only one or two studies.

3. Reliance on parental proxy perspectives

In 6 of the 17 included studies, perspectives were exclusively parental rather than directly reported by CYP. One additional study included mixed perspectives. Therefore, the abstract’s claim of examining CYP perspectives is somewhat overstated.

Recommendation: Please discuss more explicitly the methodological implications of relying on proxy perspectives. It may also be worth revisiting the wording of the abstract and potentially the title to better reflect the predominance of parental voices in part of the evidence base.

4. Selection bias and participant recruitment

Most included studies recruited participants in clinical settings or among individuals already engaged with healthcare or research systems. Participants willing to engage in focus groups or research discussions about health data are likely to have more favourable attitudes toward research participation than underserved populations more broadly.

The observed “conditional support” may therefore partly reflect selection bias rather than a robust estimate of real-world attitudes. This issue is currently underdeveloped in the manuscript.

Recommendation: Please add a more explicit discussion of potential selection bias in the Strengths and Limitations section.

Minor comments

1. Several sections of the Discussion become somewhat repetitive, particularly regarding the concept of “conditional support”. Greater synthesis and conciseness would improve readability.

2. Minor grammatical and stylistic issues:

• “data are routinely reused beyond its original purpose” → “their original purpose”

• “governance structures that is visible” → “governance structures that are visible”

3. Please ensure that abbreviations are defined at first use unless universally recognised. Some abbreviations appear before being defined (e.g., UNCRC, NIHR, FXS, ASD).

4. The statement:

“Perspectives could be reported directly by CYP, defined as individuals aged 0–25 years”

raises a conceptual question. Why were participants older than the age of legal majority included within the CYP category? A brief justification would be useful.

5. Lines 273–276 repeat content already presented in lines 169–173 regarding the NIHR-INCLUDE framework definition.

6. Line 336:

“Discrepancies were resolved through discussion.”

Was a third reviewer involved when consensus could not be reached?

7. Lines 350–352:

“Two authors (CB/LZ) appraised a subset of articles (n = 5) jointly…”

The description of how quality appraisal was subsequently divided between reviewers is somewhat unclear and may benefit from clarification.

8. There are several inconsistencies in decimal notation throughout the manuscript (commas and decimal points).

9. Line 440:

“people believed that the NHS should have unrestricted access…”

Please clarify “NHS” for international readers.

10. Line 987:

“Only studies reported in English were included…”

Please clarify whether this was due to language limitations, feasibility constraints, or lack of non-English literature identified.

11. Table 1:

“Eligibility Citeria” should read “Eligibility Criteria”.

12. No studies were excluded based on quality appraisal scores. Please discuss more explicitly how lower-quality studies may have influenced the certainty and robustness of the conclusions.

Overall, I believe this is an important and thoughtful manuscript addressing a highly relevant issue in paediatric and digital health research. With several clarifications and a more cautious interpretation of the available evidence, the paper would make a valuable contribution to the field.

**Reviewer #2:**  Thank you for your paper; it is well-prepared. I have only one recommendation to raise.

I believe that you should more clearly highlight why you focused this research on the combined population of children and young people, but not divide them into two different groups.

**Reviewer #3:**  The authors conducted a systematic review of literature to understand the attitudes of children and young people (CYP) and underrepresented groups towards big data biomedical research. They identified 17 studies in scope for this review and synthesized in x major findings: patterns around conditional support of the use of big data in pediatric healthcare research, motivations and concerns around big data, especially social and personal benefit and privacy risks, and the importance of governance structures around big data for research.

I appreciated how this work draws attention to patient perspectives that are both at once, central to the pediatric care, and simultaneously given insufficient voice to the research that could be conducted with their data. I felt there was good rigor in how systematic review process was conducted, including the use of appropriate tools for quality assessment and narrative synthesis. I also appreciated that effort was taken to differentiate attitudes between subgroups and populations (especially in lines 480, 504, 553, 608) as the authors note that underrepresented groups are heterogeneous and have intersectional identities that can influence attitudes (line 186). I also appreciated that the authors differentiate between CYP attitudes and the attitudes of representing adults (such as in line 762). I also appreciate the authors pointing out that underrepresented groups are underrepresented in both biomedical datasets and in attention towards their attitudes towards data use and researcher (line 914).

One area that this paper could be strengthened is to organize and emphasize the cross-sectional differences in the findings. This is already partially done, as noted above, but I believe reorganizing the discussion to directly address these differences would be valuable. For example, the section "Concerns and Privacy Risks" and paragraph around line 741 read as a laundry list of issues without fully examining who has which issues. By grouping everyone together, this organization undermines the authors' argument that these populations are heterogeneous and can have different concerns. I understand the limitations of the search, which did not cover a lot of the Global South, but I feel discussing these limitations alongside illustrative examples of intersectional attitudes in different contexts will do a good job of motivating the authors' call for more direct research exploring the attitudes of underrepresented populations (line 911).

I also feel that the authors should spend more time addressing the context of the included papers. While the authors point out that there are different types of underrepresented groups, each with their own potential concerns around data usage, the position of underrepresentation is context-specific. An ethnic group which is underrepresented in one context is not so in another. I discussing the findings, I would appreciate more detail about not only the nature of the group expressing the concern, but also the setting that they are in.

Along those lines, I would appreciate more reflection on the methodology of this search. In particular, it's not clear to me how the term lists for the search were constructed, and as this term list is designed to catch research about "underrepresented groups," I'm left wondering how well it does so and whether it does so from the perspective of the context of the authors. I would also appreciate some detail on who is involved in the screening and coding process and what efforts the authors made to improve the reliability of their process.

**********

what does this mean?). If published, this will include your full peer review and any attached files.

**Do you want your identity to be public for this peer review?**  If you choose “no”, your identity will remain anonymous but your review may still be made public.

For information about this choice, including consent withdrawal, please see our Privacy Policy

Reviewer #1: No

Reviewer #2: No

Reviewer #3: No

**********

**Figure resubmission:**

**Reproducibility:**

To enhance the reproducibility of your results, we recommend that authors of applicable studies deposit laboratory protocols in protocols.io, where a protocol can be assigned its own identifier (DOI) such that it can be cited independently in the future. Additionally, PLOS ONE offers an option to publish peer-reviewed clinical study protocols. Read more information on sharing protocols at https://plos.org/protocols?utm_medium=editorial-email&utm_source=authorletters&utm_campaign=protocols

# Author comment: Author response to Decision Letter 1

**DOI:** 10.1371/journal.pdig.0001434.r002

17 Aug 2026

Attachment Submitted filename: Response to Reviewers.docx

# Peer review: Decision Letter 1

**DOI:** 10.1371/journal.pdig.0001434.r003

**Authors:** Po-Chih Kuo

3 Sep 2026

Perceptions, attitudes, and concerns of underrepresented groups regarding the use of big data in children and young people’s healthcare research – a systematic literature review

PDIG-D-26-00740R1

Dear Ms Burdge,

We are pleased to inform you that your manuscript 'Perceptions, attitudes, and concerns of underrepresented groups regarding the use of big data in children and young people’s healthcare research – a systematic literature review' has been provisionally accepted for publication in PLOS Digital Health.

Before your manuscript can be formally accepted you will need to complete some formatting changes, which you will receive in a follow-up email from a member of our team.

Please note that your manuscript will not be scheduled for publication until you have made the required changes, so a swift response is appreciated.

IMPORTANT: The editorial review process is now complete. PLOS will only permit corrections to spelling, formatting or significant scientific errors from this point onwards. Requests for major changes, or any which affect the scientific understanding of your work, will cause delays to the publication date of your manuscript.

If your institution or institutions have a press office, please notify them about your upcoming paper to help maximize its impact. If they'll be preparing press materials, please inform our press team as soon as possible -- no later than 48 hours after receiving the formal acceptance. Your manuscript will remain under strict press embargo until 2 pm Eastern Time on the date of publication. For more information, please contact digitalhealth@plos.org.

Thank you again for supporting Open Access publishing; we are looking forward to publishing your work in PLOS Digital Health.

Best regards,

Po-Chih Kuo, Ph. D.

Section Editor

PLOS Digital Health

***********************************************************

**Additional Editor Comments (if provided):**

**Reviewer Comments (if any, and for reference):**

Reviewer's Responses to Questions

**Comments to the Author**

Reviewer #1: All comments have been addressed

Reviewer #3: All comments have been addressed

**********

publication criteria?

Reviewer #1: Yes

Reviewer #3: Yes

**********

3. Has the statistical analysis been performed appropriately and rigorously?

Reviewer #1: Yes

Reviewer #3: N/A

**********

4. Have the authors made all data underlying the findings in their manuscript fully available (please refer to the Data Availability Statement at the start of the manuscript PDF file)?

The PLOS Data policy

Reviewer #1: Yes

Reviewer #3: Yes

**********

5. Is the manuscript presented in an intelligible fashion and written in standard English?

Reviewer #1: Yes

Reviewer #3: Yes

**********

Reviewer #1: (No Response)

Reviewer #3: Authors have addressed my concerns.

**********

what does this mean?). If published, this will include your full peer review and any attached files.

**Do you want your identity to be public for this peer review?**  If you choose “no”, your identity will remain anonymous but your review may still be made public.

For information about this choice, including consent withdrawal, please see our Privacy Policy

Reviewer #1: No

Reviewer #3: None

**********
